# Dietary weight-management for type 2 diabetes remissions in South Asians: the South Asian diabetes remission randomised trial for proof-of-concept and feasibility (STANDby)

**DOI:** 10.1016/j.lansea.2022.100111

**Published:** 2023-02

**Authors:** Naveed Sattar, Paul Welsh, Wilma S. Leslie, George Thom, Louise McCombie, Naomi Brosnahan, Janice Richardson, Jason M.R. Gill, Luisa Crawford, Michael E.J. Lean

**Affiliations:** a School of Cardiovascular and Metabolic Health, BHF Glasgow Cardiovascular Research Centre, University of Glasgow, 126 University Place, Glasgow, G12 8TA, UK; b Human Nutrition, School of Medicine, Dentistry and Nursing, College of Medical, Veterinary & Life Sciences, University of Glasgow, Glasgow, G31 2ER, UK; c Counterweight Ltd, London, UK; d NHS Forth Valley, Stirling Community Hospital, Livilands Gate, Stirling, FK8 2AU, UK

**Keywords:** South Asian formula diet, Remission, T2D, Weight management

## Abstract

**Background:**

We aimed to assess whether a structured weight management programme incorporating a total diet replacement (TDR) (3–5 months ∼850 kcal/day formula diet) weight loss phase is acceptable to people of South Asian ethnicity and can achieve type 2 diabetes (T2D) remissions similarly to other populations.

**Methods:**

Adults of South Asian ethnicity, aged 18–65 years, with T2D for ≤4 years, and BMI 25–45 kg/m^2^ were recruited from primary care and social media, and randomised to commence TDR either immediately (iTDR), or delayed (dTDR) for 3–5 months as a usual care control arm during this period. Intervention effects were tested in randomised comparisons powered to detect significant weight loss, and in an expanded observational analysis to determine remission effect size, including both iTDR and dTDR groups. Acceptability in those recruited was explored by questionnaire and weight change.

**Trial registration:**

Current Controlled Trials, ISRCTN10720065. Date of Registration 27/09/2017.

**Findings:**

Twenty-five eligible individuals were recruited. Mean baseline (SD) age was 45.8 (11.1) years, weight 88.2 (13.7) kg, BMI 32.1 (3.8) kg/m^2^, HbA1c 60.4 (11.3) mmol/mol, liver fat by MRI 15.6 (9.4)%. In the RCT, mean(SD) weight change after TDR was −7.7 (7.2)% in the intervention group (n = 13), and −1.2 (1.4)% in the usual-care control group (n = 12) (p = 0.005), with T2D remission achieved by 5/13, compared to 0/12 respectively (p = 0.039). In the observational study, 23/25 started TDR and 19/23 participants completed the TDR phase. Median time spent in TDR was 105 days (IQR 77–134 days). T2D remission was achieved in 10/23 (43%), and weight changes were concordant with the RCT. Overall, 8/23 (35%) lost over 10% bodyweight. Absolute liver fat proportion near halved from 15.3% at the start of TDR to 8.6% (p < 0.001).

**Interpretation:**

In UK-based South Asians, TDR-led weight loss and T2D remission rates are comparable to those observed in white cohorts, and the intervention was acceptable in most of those recruited. There is potential to further improve outcomes, but one-third lost >10% body weight, and the mechanism underpinning T2D remission appears similar, driven by weight change with loss of excess ectopic body-fat.

**Funding:**

We gratefully acknowledge funding for the MRI scans from the, Miss MJM Smith Trust (registered charity: SC040586). No other external funds were provided for this trial. NS is supported by the British Heart Foundation Research Excellence Award (RE/18/6/34217).

**Research in context**

**Evidence before this study**

Two major randomised trials of type 2 diabetes remission have been published, one in the UK (DiRECT) among predominantly white Europeans and one from Qatar in Middle Eastern people (DIADEM-I). Both had striking results, which have influenced several relevant clinical guidelines. However, despite some anecdotal or observational evidence, randomised trials testing for remission of diabetes by weight loss are lacking in South Asians, a group that forms 24% of the world's population. Such evidence is critical, as it is not known if this form of diabetes, which takes an aggressive course at lower BMI, can be reversed into remission by weight loss. It is also not known whether liver fat, known to be higher in South Asians and causally linked to diabetes in Europeans, is also reduced by weight loss in South Asians.

STANDby tested the same weight management programme as in DiRECT but employed a more economical study design. An initial RCT was powered to investigate weight change efficacy, assuming a large effect size based on DIRECT and DIADEM-I that found 3–4-month weight change highly predictive of 12-month data. Delayed treatment was undertaken for the RCT control group, firstly to enhance participant retention in the control group, and secondly to increase numbers with which to estimate remission effect-size.

**Added value of this study**

Despite being conducted during the COVID-19 pandemic with all its obstacles to research, incurring need for protocol modifications, STANDby generated encouraging data to show that weight loss can lead to remission of type 2 in South Asians, accompanied by loss of excess liver fat measured by MRI. The innovative study design in STANDby involved a sensible (pandemic adjusted) duration for the primary outcome, remission at ∼4 months, which clearly captures the treatment effect, and which allowed the control participants then to receive the intervention. This gave them a desired, valued, treatment—thus enhancing participant retention. It also provided greater numbers for estimation of effect size. The results from the RCT and enlarged observational group were concordant. These findings confirm that a diet programme with an initial 850 kcal/day formula-diet phase can generate weight losses and type 2 diabetes remission rates in UK South Asians comparable with UK whites, and that the intervention appeared acceptable to recruited South Asians.

**Implications of all the available evidence**

Intentional weight loss purely by a hypocaloric dietary intervention leads to remission of type 2 diabetes for large numbers of people with early onset disease in all ethnic groups studied to date. Likelihood of remission across all randomised trials appears directly proportional to degree of weight loss achieved within the trial, with just under 5% remission for every 1% weight loss within the first 6 years of diagnosis. These findings further suggest type 2 diabetes is a disease mediated by ectopic fat excess, reversible with weight loss. We need further larger, longer, pragmatic studies to see how we can increase acceptability of this approach in South Asians, and whether substantial weight loss with remission of diabetes can be sustained in large numbers using tailored methods. Such studies, which could be well-designed pragmatic observational studies rather than trials, should now be promoted more aggressively on the back of the present proof-of-concept data.

## Introduction

Globally, type 2 diabetes (T2D) affects over 400 million people, and around one in ten adults in the UK.^1^ Around one in four people worldwide are of South Asian origin, with considerably higher risk of T2D than the general UK/European white population, developing the condition at a lower body mass index and at younger ages.^2^ The mechanism for their excess risk likely includes greater total fat mass and lower lean mass, leading, in turn, to more rapid ectopic fat gain when weight increases,^3^ and potentially lower beta cell reserve.^4^ Lack of South Asians in the DiRECT trial, and continuing uncertainty over detailed mechanisms behind excess diabetes risk in Souths Asians, have contributed to reluctance to offer remission by dietary means. Furthermore, recent qualitative research suggested that total dietary replacement (TDR) approach might be less acceptable to South Asians seeking T2D remission, and sub-optimal engagement, and outcomes with weight management among South Asians.^5^, ^6^, ^7^ Few Asians, the largest UK ethnic minority,^6^ or their health care workers, prioritise effective weight management or seek remission of T2D by weight loss. Without appropriate research and development, their T2D rates and early onset of complications will continue to rise.

Our recent work^8^ showed how weight loss of around 10 kg, using an evidence-based weight management programme, ‘Counterweight-Plus’, resulted in remission of diabetes at one year in 46% of people with diagnosed T2D of less than six years' duration. The programme induces weight loss with a period of formula low energy TDR, providing all essential nutrients in around 850 kcal/day, creating a sizable energy deficit and therefore substantial weight loss. We also showed diabetes remission was accompanied by sizeable reductions in liver fat content,^9^ as an important likely causal mechanism of benefit.^10^

In DiRECT, almost all participants were white British, so doubts remained about extrapolating its results to other ethnic groups. The present study therefore examined whether the initial phase of the Counterweight-Plus programme (TDR and Food Reintroduction (FR)) was acceptable to the South Asian population and if it induced similar clinical outcomes in terms of weight loss, liver fat reduction, and remission of diabetes. The results, if positive, should have far-reaching consequences for a large proportion of the world's diabetes population, to contribute to development of culturally optimal practical approaches for T2D remission.

## Methods

### Design and participants

**Fig. 1:**
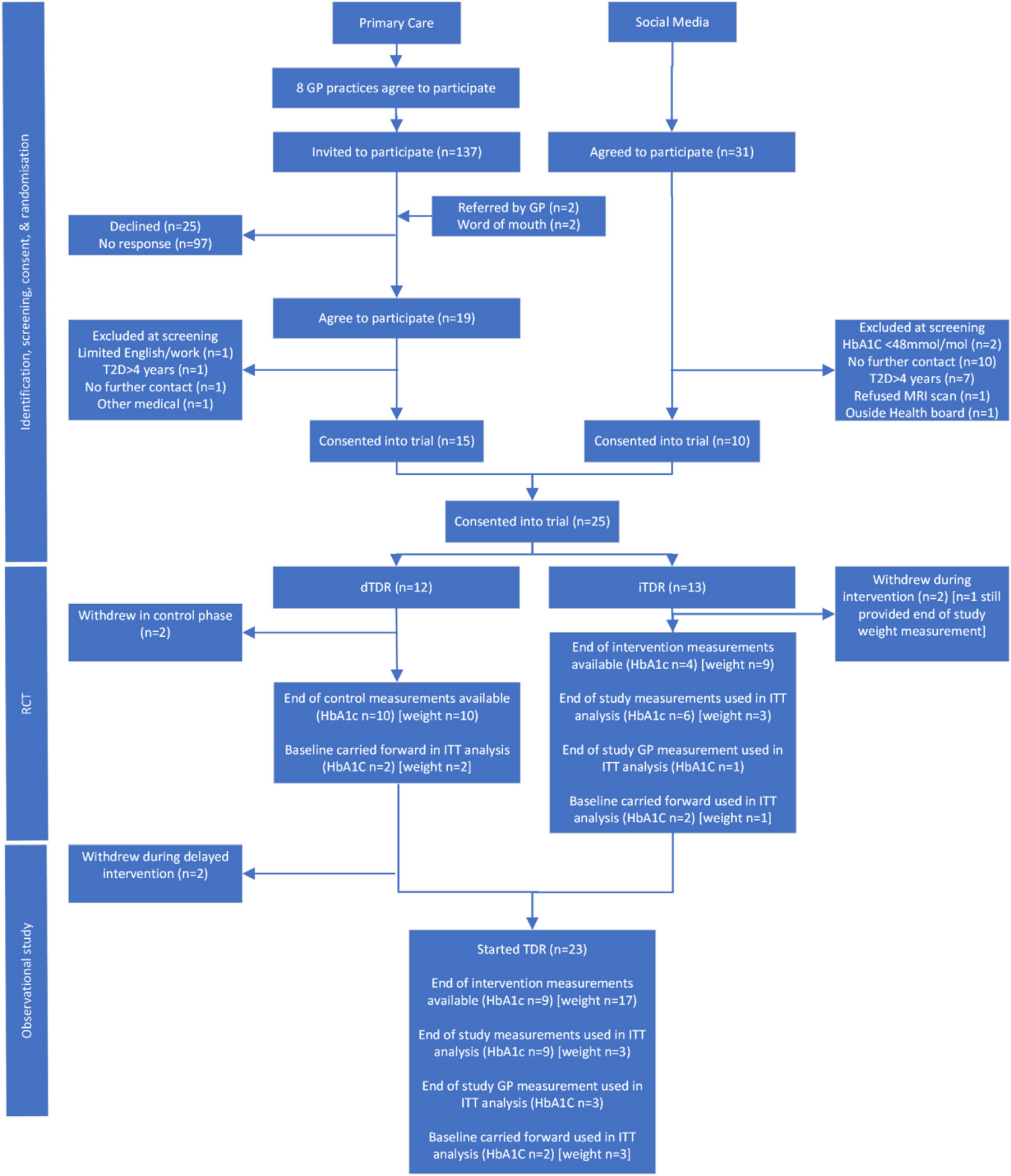
Flow chart of recruitment, retention, and data availability in the randomised controlled trial and observational study.

This work encompassed (i) a randomised clinical trial as proof of concept, (ii) extended observational follow-up in larger numbers to strengthen the proof of concept with observational programme acceptability assessment in all participants (Fig. 1). Participants were randomised either to commence the intervention (Counterweight-Plus) immediately after randomisation (immediate intervention group [iTDR]), or to continue usual management of T2D for three months, prior to starting the intervention (control/delayed intervention group [dTDR]). This study design provided comparisons between groups during the randomised phase, and observational data in an enlarged cohort (iTDR plus dTDR) following intervention. The objective was to assess whether offering patients of South Asian ethnicity a structured weight management programme (Counterweight Plus) with primary focus on the initial Total Diet Replacement phase (TDR), will achieve substantial weight change and remission of diabetes in this population, as published in other populations.^11^

Primary care practices in areas with known South Asian communities were invited by e-mail to participate by identifying potential participants by a computerised search of GP records. Inclusion criteria were men and women aged 18–65 years, South Asian ethnicity, T2D of duration 0–4 years (diagnosis based on a recorded diagnostic test), HbA1c ≥48 mmol/mol and/or fasting plasma glucose ≥7 mmol/l within last 12 months, body mass index (BMI) >25 kg/m^2^ and <45 kg/m^2^ (upper cut-off to permit MRI scanning). Lists generated by the search were screened by GPs to remove individuals unsuitable for the trial. Potentially eligible individuals were mailed an invitation to participate, with an information sheet, and asked to respond using prepaid reply envelopes. Reminders were sent to those who did not respond. The study was also advertised via media platforms (radio, social media). The advertisement provided information on the eligibility criteria and contact details for the study team to discuss participation in more detail. Individuals interested in participating attended an initial screening appointment with a research dietitian, to explain the study, check eligibility, provide samples of the formula diet, and agree participation. Written informed consent was secured from all participants. Ethical approval was granted on 7th June 2017 (West of Scotland REC 3 17/WS/0104), and NHS Greater Glasgow and Clyde R&D approval was granted. All subsequent protocol amendments were similarly approved.

### Randomisation and masking

Participants were randomised after informed consent was taken, using a blinded envelope system prepared independently of the research team. Research dietitians enrolled participants, and assigned them to the allocated interventions. Given the nature of the intervention, participants and research dietitians delivering the intervention and collecting outcome data were aware of treatment allocation. However, the laboratory staff and statisticians were blind to the randomisation allocation.

### Procedures

Participants randomised to iTDR commenced the Counterweight Plus programme which was used in the DiRECT trial.^8^ Participants randomised to dTDR were advised to continue with usual care, managed under current NHS clinical guidelines, for three months before returning to receive the intervention. They were asked not to aim to lose any weight during the initial delay/control period, but rather to wait until they commenced the intervention.

The intervention, delivered by the study research dietitians experienced in Counterweight Plus programme delivery, comprised a Total Diet Replacement phase (TDR: an 825–853 kcal/day formula diet) for 3–5 months (flexible duration to accommodate participants with commitments or practical limitations due to COVID-19), and then stepped food reintroduction (FR) for six-eight weeks. On completion of FR period participants were given advice, and written information, on maintaining weight loss, and returned to the care of their GP, who was informed of the trial and their patient's progress. Oral diabetes medications, antihypertensive, and diuretic drugs were withdrawn on commencement of TDR and reintroduced (according to NHS clinical guidelines) if diabetes or hypertension returned.^12^^,^^13^

Under the intended design of the study, participants were expected to attend the Glasgow Clinical Research Facility (CRF) for all appointments and be seen for review one week after commencing TDR, and at two weekly intervals thereafter. Participants randomised to iTDR had a randomisation appointment where bloods were drawn, with further intended blood draws after completion of TDR and FR phases. Participants delayed to dTDR were intended to have an additional appointment for biochemistry after the end of the control phase. All participants were intended to have whole-body MRI at baseline, after TDR, and after FR.

#### COVID-19-related protocol amendments

During the periods with *COVID-19* related lockdowns and restrictions, the protocol was adapted or curtailed, at different stages for different participants, whilst retaining focus on obtaining results, which would satisfy the primary purpose of the study. These COVID-engendered design modifications were of course inevitably post hoc but made best use of the hard work of the participants and the research team under extremely difficult circumstances, such that the results remained scientifically valid. Recruitments entailed face-to-face interviews at the CRF with the research dietitian and were suspended from 16th March to 24th July 2020. Intervention review appointments/consultations, for participants already recruited, were carried out by the research dietitian, by telephone/text/e-mail, as preferred by the participant. Follow up contact, for participant safety and outcome assessments (HbA1c, fasting blood glucose, and blood pressure), was required at the end of the TDR and at the end of the study. Participants unable or unwilling to attend the CRF were asked to attend their GP practice for these measurements. Due to these necessary pandemic-related amendments, weight, HbA1c, and other blood measurements were not uniformly completed by all participants at all appointments, and the availability of scanners for research, thus timings of study MRI scans, varied considerably. Therefore, the analysis schedule was modified to optimise use of available data. Analysis of changes at the end of FR, as a separate time point, was eliminated. Instead, the earlier post-control/post-TDR measurements of blood biomarkers and weight were considered the primary time point for outcome evaluation, and study measurements from end of FR were used as an outcome measurement where no measurement at the end of TDR was available. GP blood tests for HbA1c were used to replace central lab measurements where no blood sample was available. Where data at neither time point was available, baseline-carried-forward was used for a highly conservative intention to treat (ITT) analysis (Fig. 1). To justify the approach that end of FR (end of study) HbA1c is a reasonable alternative for HbA1c at 3-months/end of TDR, because HbA1c changes slowly after a change in glycaemia, a post-hoc validation analysis was carried out, comparing end of TDR HbA1c with end of study HbA1c in participants with available paired measurements (n = 8). The end of study measurement was not statistically significantly different from the end of TDR measurement (mean difference +2.0 mmol/mol at the end of FR (95% −1.1, 5.2) p = 0.17).

### Outcome measures

The main outcomes in this proof of principle study were numbers recruited to participate in the intervention, programme retention, acceptability of the intervention to volunteers of SA ethnicity by questionnaire, weight loss achieved (absolute kg, percentage weight change, and proportion achieving ≥15 kg weight loss), and of T2D (defined as HbA1c <48 mmol/l off diabetes treatment^14^ for at least 3 months). Other exploratory outcomes included change in HbA1c, change in other blood biomarkers (as described below), and change in liver fat and visceral fat mass. Whole-body MRI scans were carried out at the Clinical Research Imaging Facility, Queen Elizabeth University Hospital, Glasgow using a 3.0 T Prisma magnetic resonance scanner with a dual-echo Dixon Vibe protocol (Siemens, Munich, Germany).^15^ Anonymised digital data derived from the scanning procedure, were sent by secure transfer to Advanced MR Analytics (AMRA) for analysis. Outcome measurements from MRI included total adipose tissue volume, visceral adipose tissue volume, lean tissue volume, and liver fat percentage.

Fresh blood samples were transferred to a central laboratory for processing within 2 h of collection, and blood sample aliquots were then frozen at −80 °C until assay of all samples at the end of the trial. A central laboratory measured HbA1c, liver function tests (AST, ALT GGT), total cholesterol, HDL-cholesterol, triglycerides, glucose, high sensitivity C-reactive protein, ferritin, urea, urate, creatinine, and insulin were measured using automated platforms and the manufacturer's calibrators and quality controls (e411 and c311, Roche Diagnostics, Burgess Hill, UK).

### Sample size

Based on the power calculation, the study aimed to recruit 25 participants. Anticipating a failure to complete rate of 25% (estimated before the COVID-19 pandemic), 20 participants in a randomised comparison, provided power to estimate the change in weight achieved by the intervention with a margin of error of 2.8 kg assuming a standard deviation weight change over 3 months of 6 kg (the amount observed in the DiRECT trial) For example, for an average observed weight change of 8 kg (SD 6) we can estimate with 95% confidence that in the population the true weight change would lie between 5.1 and 10.7 kg. The proportion achieving remission in expanded observational cohort (assumed n = 20) was powered for comparison with the proportion achieving diabetes remission in the external DIADEM-I randomised controlled trial control group (12%; 95% CI 5%, 21%).^11^ Therefore assuming 12% remission in a usual care group (based on DIADEM) with 20 participants ultimately completing TDR there would be 83% power to detect a difference of at least 30% in participants (n = 6) achieving remission.

### Statistical analyses

Recruitment, retention, and acceptability of the intervention were reported using descriptive analyses: postal acceptances as a proportion of invitation letters sent, consenting study participants, proportion of randomised participants withdrawing, proportion of expected meal replacements dispensed. Acceptability of the intervention indicated by questionnaire was explored using raw questionnaire responses.

The randomised analyses, and the expanded observational analyses combining iTDR and dTDR groups, were conducted using a modified intention to treat (ITT) analysis (Fig. 1). A per protocol (PP) analysis did not use baseline carried forward, and treated participants lacking both the end of TDR/control and end of FR measurements as missing from analyses for that variable.

Baseline variables, change in outcome variables from baseline following the initial RCT, and change in outcome variables from baseline following the intervention (observational study), were summarised in both ITT and PP analyses. Categorical variables are reported as a number (%), and tests for differences across randomised groups using Fishers exact test or Chi-squared test. Continuous variables were summarised as mean (standard deviation) for normally distributed variables and median and inter-quartile interval (IQI) for skewed variables. Tests of significance of the effect of the intervention were analysed by two-tailed t-test (two sample t-test in the randomised analyses and one sample t-test in the observational analyses). In the observational analyses, weight observed over weeks in the study was modelled using a mixed model, stratified by iTDR or dTDR randomisation, with week zero being the baseline and week 13 being the end of study appointment. In the observational study, changes in categorical variables were tests using McNemar's test. Change in weight was modelled as an outcome variable in univariable linear regression, with explanatory variables being change in other variables of interest. All analyses were conducted in STATA version 17.0.

### Role of the funding source

The funders had no role in the study design; in the collection, analysis, and interpretation of data; in the writing of the report; and in the decision to submit the paper for publication.

## Results

### Recruitment, randomisation, and retention

Recruitment commenced on 2nd Feb 2019 and was completed on 30th July 2021, with a period of recruitment suspension due to the first COVID-19 lockdown. Following meetings with the study team, eight out of 16 invited practices agreed to participate. From these practices, 137 potentially eligible people were mailed an invitation to participate (Fig. 1).

Fifteen people (11.8%) replied accepting the invitation. Two more people fulfilling the study criteria were referred to the study team by their GP, and a further two self-referred to the study team after hearing about the study. Of these 19 people, four were excluded at screening (Fig. 1), and therefore a total of 15 people recruited via Primary Care were consented into the trial. Thirty-one people responded to media advertisements, expressing interest in participating. Of these twenty-one were excluded on screening for inclusion criteria, and ten were consented into the trial (Fig. 1).

Overall, 13 participants were allocated to iTDR, and the remaining 12 to dTDR. In total, six participants withdrew during the study. Two withdrew from the dTDR group during the initial control period citing “change of mind” as the reason for withdrawal (Fig. 1). Since these participants started the RCT but not the TDR, they were included in the ITT analysis for the randomised comparison, but not the subsequent observational study of TDR effect (Fig. 1). Four participants withdrew after commencing TDR, citing: illness, work commitments/unable to follow a TDR, social issues, and COVID-19 infection.

### Baseline

Mean age at recruitment was 45.8 years (standard deviation (SD) 11.1 years) and 52% were male. Mean BMI was 32.1 kg/m^2^ (SD 3.8 kg/m^2^). Liver fat was generally high at mean 15.6% (SD 9.4%). Mean duration of T2D was 1.9 years since diagnosis. Most participants were prescribed glucose-lowering medication (14/25, 56%) (Table 1), among whom 2/14 were on two or more drugs, and some were newly diagnosed. Only 3/25 were current smokers (12%). Six participants (24%) reported engaging in recent/current weight management activities. The study population had no medical history of cardiovascular disease (no myocardial infarction, angina, stroke, transient ischaemic attack, heart failure, peripheral vascular disease, or atrial fibrillation), four had a history of hypertension, and three were on antihypertensive medication. There was generally good balance between the randomised groups (Table 1).

**Table 1:** Baseline characteristics of study population at the start of the randomised controlled trial.

Factor	Overall (n = 25)	dTDR (n = 12)	iTDR (n = 13)
Age, mean (SD)	45.8 (11.1)	44.3 (11.9)	47.2 (10.7)
Male sex	13 (52%)	5 (42%)	8 (62%)
Current smoker	3 (12%)	1 (8%)	2 (15%)
Height (cm)	165.6 (9.5)	165.9 (9.2)	165.3 (10.1)
Body mass index (kg/m^2^)	32.1 (3.8)	31.6 (3.3)	32.6 (4.4)
Baseline weight (kg)	88.2 (13.7)	87.0 (10.1)	89.3 (16.7)
Hypertension	4 (16%)	2 (17%)	2 (15%)
Arthritis	1 (4%)	0 (0%)	1 (8%)
Hyperlipidaemia	6 (24%)	2 (17%)	4 (31%)
Prescribed antidiabetic medication at baseline	14 (56%)	9 (75%)	5 (38%)
Prescribed antihypertensive medication at baseline	3 (12%)	2 (17%)	1 (8%)
Taking part in other weight management activities	6 (24%)	2 (17%)	4 (31%)
Waist circumference (cm)	104.4 (6.0) (n = 23)	105.7 (4.1)	102.9 (7.4) (n = 11)
HbA1c (mmol/mol)	60.4 (11.3)	64.3 (14.2)	56.8 (6.5)
SBP (mmHg)	132.0 (15.8)	135.5 (18.0)	128.9 (13.4)
Glucose (mmol/L)	8.03 (2.52)	8.75 (2.99)	7.36 (1.88)
Total cholesterol (mmol/L)	4.48 (1.20)	4.48 (0.90)	4.48 (1.47)
HDL-cholesterol (mmol/L)	1.07 (0.22)	1.12 (0.24)	1.03 (0.19)
Triglycerides (mmol/L)	1.74 (1.19, 2.72)	1.50 (1.15, 3.00)	1.77 (1.26, 2.29)
Urea (mmol/L)	3.96 (0.96)	4.13 (1.17)	3.80 (0.73)
Uric acid (mg/dL)	4.98 (1.18)	5.02 (1.35)	4.94 (1.05)
Estimated glomerular filtration rate (ml/min/1.73 m^2^)	118.8 (14.7)	118.2 (17.4)	119.3 (12.2)
GGT (U/L)	34 (23, 51)	47 (32, 88)	24 (20, 34)
ALT (U/L)	27 (17, 33)	30 (26, 42)	20 (16, 30)
AST (U/L)	18 (17, 24)	23 (18, 28)	17 (14, 18)
C-reactive protein (mg/L)	4.30 (1.61, 7.18)	3.01 (1.37, 5.88)	7.09 (1.61, 8.85)
Ferritin (ug/L)	84 (52, 151)	79 (54, 153)	96 (52, 127)
Insulin (uU/ml)	23.9 (17.9, 26.9)	24.6 (17.3, 29.8)	22.3 (17.9, 26.4)
HOMA-IR	7.7 (5.6, 10.5)	8.7 (6.3, 12.6)	6.5 (5.6, 8.2)
EQ5D (VAS units)	54.8 (22.7)	50.3 (22.2)	58.9 (23.3)
**MRI derived anthropometrics**			
Total adipose tissue volume (L)	29.3 (8.1)	27.9 (5.8)	30.6 (9.8)
Abdominal subcutaneous adipose tissue (L)	10.9 (4.0)	10.6 (3.3)	11.2 (4.7)
Lean volume (L)	21.4 (4.0)	21.7 (3.9)	21.2 (4.3)
Liver fat (%)^a^	15.6 (9.4)	15.1 (10.2)	16.1 (9.0)
Visceral adipose tissue volume (L)	5.1 (1.2)	5.1 (1.3)	5.1 (1.1)

Data are count (%), mean (SD), or median (IQI).

a One observation based on follow-up appointment carried backwards in the dTDR group.

### Randomised controlled trial

Median time spent in TDR in the iTDR group was 105 days (IQR 87–130 days). Median time in the control phase in the dTDR group was 104 days (IQI 101–108 days).

#### Weight

Among the iTDR group, n = 9 weight measurements were available immediately after TDR, and n = 3 weight measurements were collected after FR including one voluntary measurement from a participant who withdrew from iTDR (median for the n = 3: 170 days after completing TDR, range 97–270). In the control dTDR group, n = 10 weight measurements were collected immediately after the control period (Fig. 1).

#### HbA1c

Among the intervention group, n = 4 HbA1c measurements were available immediately after TDR, with n = 7 HbA1c measurements collected after FR (median for the n = 7: 150 days after completing TDR, range 41–227). In the control dTDR group n = 10 HbA1c measurements were collected immediately after the control period.

#### MRI

Overall, n = 20 participants had MRI data available after the RCT (n = 9 in dTDR, n = 11 in iTDR), with n = 13 participants' measurements taken within ±2 weeks of the end of the RCT phase. The median time between end of the RCT and MRI measurement was 5.5 days (range −13, 231 days).

### Randomised Comparison

At the end of the RCT, mean weight change from baseline was −0.9 kg (SD 1.2 kg) [−1.2% (SD 1.4%)] in the usual-care control group, and −7.2 kg (SD 7.8 kg) [−7.7% (SD 7.2%)] in the intervention group (difference between changes −6.3 kg (95% CI −11.0 kg, −1.6 kg) p = 0.011; −6.5% (95% CI −10.9%, −2.1%) p = 0.005) (Table 2). Six participants (46%) in the iTDR group lost >10% of their body weight (Table 2), and one participant lost >15 kg. Mean HbA1c change was −0.7 (5.6) mmol/mol in the usual-care control group, and (after stopping glucose-lowering medication) −5.5 (11.0) mmol/mol in intervention group (p = 0.20 for difference in change between groups) (Table 2)

**Table 2:** Change in outcome variables during the RCT (ITT analysis).

Factor	Change in dTDR (control)	Change in iTDR (intervention)	p-value	Between group change (95% CI)
**Number analysed**	12	13		
Weight (kg)	−0.9 (1.2)	−7.2 (7.8)	0.011	−6.3 (−11.0, −1.6)
Weight (%)	−1.2 (1.4)	−7.7 (7.2)	0.005	−6.5 (−10.9, −2.1)
Weight change category				
<5% weight loss	12 (100%)	5 (38%)	0.004	–
5–9.9% weight loss	0 (0%)	2 (15%)		–
>10% weight loss	0 (0%)	6 (46%)		–
Remission	0 (0%)	5 (38%)	0.039	–
HbA1c (mmol/mol)	−0.7 (5.6)	−5.5 (11.0)	0.20	−4.7 (−12.0, 2.6)
Taking antidiabetic medication	9 (75%)	3 (23%)	0.017	–
Taking antihypertensive medication	4 (33%)	1 (8%)	0.16	–
Waist circumference (cm)	0.2 (4.8)	−4.8 (5.7)	0.027	−5.0 (−9.3, −0.6)
SBP (mmHg)	−5.1 (17.4)	−3.9 (11.1)	0.84	1.2 (−10.7, 13.2)
Glucose (mmol/L)	−0.5 (1.68)	−1.0 (1.5)	0.41	−0.5 (−1.9, 0.8)
Total cholesterol (mmol/L)	0.0 (0.4)	−0.1 (1.1)	0.91	0.0 (−0.7, 0.6)
HDL-cholesterol (mmol/L)	−0.03 (0.14)	−0.02 (0.18)	0.83	−0.0 (−0.1, 0.1)
Triglycerides (mmol/L)	−0.28 (0.71)	−0.03 (1.4)	0.59	0.2 (−0.7, 1.2)
Urea (mmol/L)	0.1 (1.1)	0.2 (1.1)	0.82	0.1 (−0.8, 1.0)
Uric acid (mg/dL)	−0.3 (0.6)	0.1 (1.2)	0.34	0.4 (−0.4, 1.2)
Estimated glomerular filtration rate (ml/min/1.73 m^2^)	1.1 (5.9)	−0.5 (10.4)	0.65	−1.6 (−8.7, 5.5)
GGT (U/L)	−16 (67)	−3 (14)	0.47	13 (−25, 53)
ALT (U/L)	−2 (7)	−2 (11)	0.97	0 (−8, 8)
AST (U/L)	−1 (4)	1 (7)	0.31	2 (−2, 7)
C-reactive protein (mg/L)	−0.8 (2.2)	−2.7 (5.6)	0.27	−1.9 (−5.5, 1.6)
Ferritin (ug/L)	−8 (17)	5 (33)	0.25	12 (−10, 35)
Insulin (uU/ml)	−2.4 (8.1)	−7.9 (15.3)	0.27	−5.6 (−15.8, 4.7)
HOMA-IR	−1.9 (4.5)	−3.3 (5.2)	0.48	−1.4 (−5.4, 2.7)
Total adipose tissue volume (L)	−0.5 (0.7)	−4.3 (4.5)	0.008	−3.8 (−6.5, −1.1)
Abdominal subcutaneous adipose tissue (L)	−0.13 (0.45)	−1.63 (1.98)	0.018	−1.5 (−2.7, −0.3)
Lean volume (L)	0.0 (0.4)	−0.8 (1.0)	0.029	−0.7 (−1.4, −0.1)
Liver fat (%)	−1.4 (3.1)	−7.0 (8.1)	0.035	−5.6 (−10.8, −0.4)
Visceral adipose tissue volume (L)	−0.1 (0.3)	−1.0 (0.8)	0.001	−0.9 (−1.4, −0.4)

Data for within group changes are count (%) or mean (SD).

Of the five iTDR group participants treated with glucose-lowering medications, which were stopped at baseline, three resumed pharmacotherapies before the study end. Of the eight iTDR group participants not treated with glucose-lowering medications, none started before study end. There were no diabetes medication changes in the dTDR control group. No participants in the iTDR group commenced new antihypertension medications, but two participants in the dTDR group did commence new antihypertension medications during the control phase.

Remission of T2D was achieved by none of the control group, compared to 5/13 (38%) in the immediate intervention group (p = 0.039).

Liver fat changed −1.4% in the control group and −7.0% in the iTDR group, between group difference −5.6% (−10.8, −0.4; p = 0.035). Other outcomes that were changed by the intervention include waist circumference, total adipose tissue, visceral adipose tissue, abdominal subcutaneous adipose tissue, and lean volume (Table 2). Results were similar in a per protocol analysis (Supplementary Table S1).

No adverse events were reported in the control group, with headache the most common adverse event in the intervention group (n = 4 (31%)) (Supplementary Table S2).

### Observational analyses: Combined data for iTDR and dTDR groups

Among 23 participants who ultimately started TDR in the enlarged observational study, median time spent in TDR was 105 days (IQR 77–134 days). Out of 23 participants who ultimately started TDR in the enlarged observational study, follow-up body weight and HbA1c were obtained immediately after completing TDR in n = 17 and n = 9 participants, respectively (Fig. 1). The analysis further included COVID-delayed measurements, collected after FR, for body weight, including one voluntary weight measurement from a participant who withdrew from TDR (n = 3: measured median 170 days after completing TDR, range 97–270 days after completing TDR), and for HbA1c measurements, (n = 10: measured median 141 days after completing TDR, range 41–227). Overall, n = 18 participants had MRI data available after TDR, with n = 11 participants’ measurements taken within ±2 weeks of the end of TDR. The median time between end of the TDR and MRI measurement was 47 days (range −8, 231 days).

**Fig. 2:**
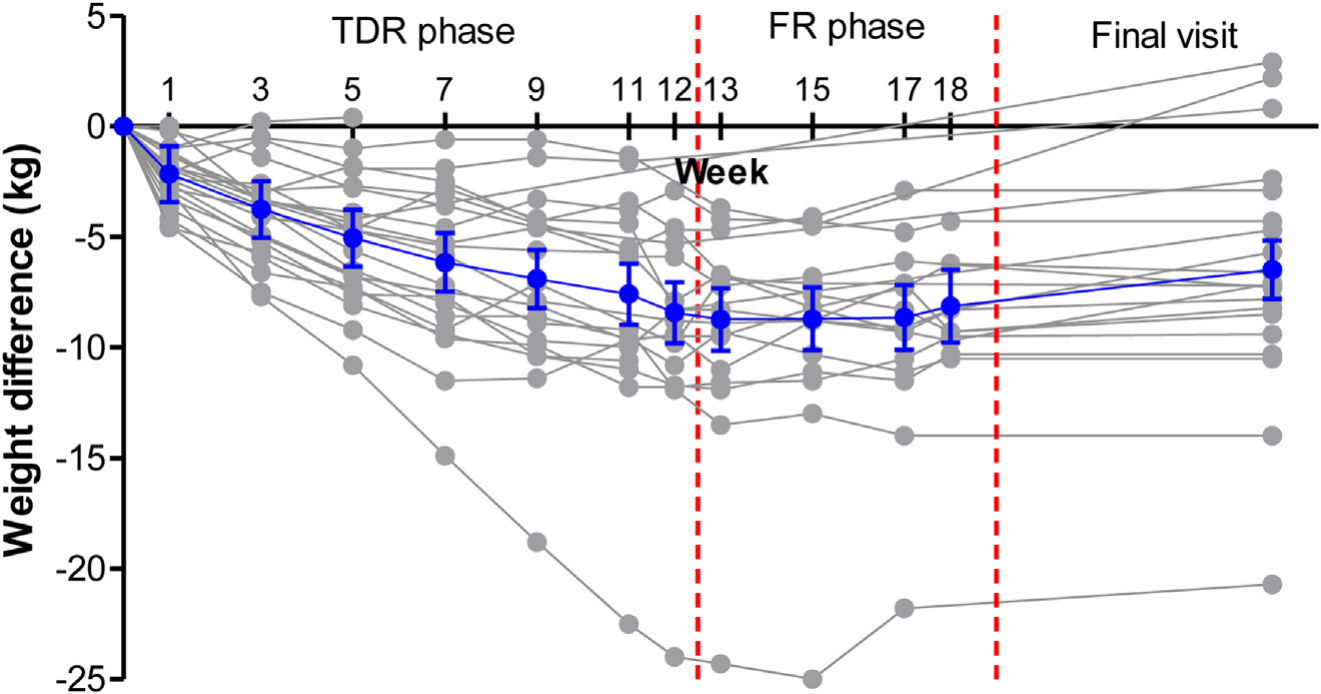
Weight change from first TDR visit to final study appointment in 23 participants who commenced TDR using all available data (grey). Overall mean change and 95% (blue) based on a mixed model.

During TDR, there was a steady reduction in weight across study appointments, plateauing around week eight (Fig. 2). The mean weight change was −6.8 kg (95% CI −9.5, −4.0 kg) equating to −7.6% (95% CI −10.4, −4.8) (Table 3). Overall, 8/23 (35%) lost more than 10% bodyweight, and one participant lost >15 kg. There was a significant reduction in HbA1c, of −7.2 mmol/mol (95% CI −12.4, −2.0 mmol/mol) achieved despite withdrawing glucose-lowering medication when starting TDR. Remission of T2D was achieved by 10/23 participants (43%, 95% CI 23–64%) after TDR. In a pre-specified analysis, this was greater than the 12% remission achieved in the DIADEM RCT control arm (p < 0.001). Absolute liver fat proportion near halved from 15.3% at the start of TDR to 8.6% (reduction of −6.7% (−9.8, −3.7%; p < 0.001)) (Table 3).

**Table 3:** Observational study of change in outcome variables between start and end of TDR and in all participants (combined iTDR & dTDR) (n = 23, ITT analysis).

Factor	Change	Lower 95% CI	Upper 95% CI	p-value
Weight (Kg)	−6.8	−9.5	−4.0	<0.001
Weight (%)	−7.6	−10.4	−4.8	<0.001
Weight change category n (%)				<0.001
<5% weight loss	9 (39%)	19%	59%	
5–9.9% weight loss	6 (26%)	8%	44%	
>10% weight loss	8 (35%)	15%	54%	
Remission n (%)	10 (43%)	23%	64%	<0.001
HbA1c (mmol/mol)	−7.2	−12.4	−2.0	0.009
Net change in taking antidiabetic medication	−6 (−26%)	−52%	0%	0.07
Net change in taking antihypertensive medication	+1 (+4%)	−15%	+23%	0.99
Waist circumference (cm)	−5.3	−7.9	−2.7	<0.001
SBP (mmHg)	−5.3	−12.3	1.8	0.14
Glucose (mmol/L)	−1.2	−2.0	−0.3	0.008
Total cholesterol (mmol/L)	−0.3	−0.7	0.2	0.24
HDL-cholesterol (mmol/L)	−0.1	−0.1	0.0	0.30
Triglycerides (mmol/L)	−0.1	−0.6	0.4	0.65
Urea (mmol/L)	0.1	−0.4	0.6	0.65
Uric acid (mg/dL)	0.2	−0.3	0.6	0.45
Estimated glomerular filtration rate (ml/min/1.73 m^2^)	−0.4	−4.8	3.9	0.83
GGT (U/L)	−10.3	−22.5	1.8	0.09
ALT (U/L)	−3.4	−8.3	1.4	0.16
AST (U/L)	−0.2	−3.2	2.8	0.88
C-reactive protein (mg/L)	−2.0	−3.9	−0.1	0.04
Ferritin (ug/L)	2.0	−10.4	14.3	0.75
Insulin (uU/ml)	−6.4	−11.9	−0.9	0.02
HOMA-IR	−2.8	−4.8	−0.8	0.007
Total adipose tissue volume (L)	−3.9	−5.5	−2.2	<0.001
Abdominal subcutaneous adipose tissue (L)	−1.4	−2.1	−0.7	<0.001
Lean volume (L)	−0.5	−0.9	−0.1	0.029
Liver fat (%)	−6.7	−9.8	−3.7	<0.001
Visceral adipose tissue volume (L)	−1.0	−1.3	−0.6	<0.001
EQ5D (VAS units)	11.2	1.9	20.5	0.02

Of the 12/23 participants who were taking diabetes medication before starting the study, and then stopped prior to commencing TDR, seven stayed off diabetes medication after completing TDR, and five restarted pharmacotherapies. Among the 11/23 who were not taking diabetes medication at baseline, one commenced pharmacotherapy after completing TDR.

Of 3/23 participants three taking antihypertensive medications at baseline, two recommenced after TDR. Of the 20 participants not taking antihypertensive medications at baseline, two commenced taking medication after TDR.

The intervention was also associated with significant reductions in waist circumference, glucose, insulin, and HOMA, CRP, total adipose tissue, and visceral adipose tissue, abdominal subcutaneous adipose tissue, and lean body volume. Quality of life by EQ5D increased by 11.2 (95% CI 1.9, 20.5) following intervention (Table 3). These results were all similar in a PP analysis (Supplementary Table S3).

In the ten participants who achieved remission in the combined analysis, average weight loss was −9.5 (SD 7.0) kg (−10.8 (SD 6)%), compared to −4.7 kg (SD 5.2) and −5.2% (SD 5.9%) in those who did not achieve remission (p = 0.074 for absolute change and p = 0.035 for percentage change). Among the ten participants who achieved remission, liver fat decreased by −7.6%, from mean 11.8% at baseline to mean 4.2% after the intervention. Among the 13 participants who did not achieve remission, liver fat decreased by −6.0%, from mean 18.0% at baseline to mean 12.0% after the intervention (p = 0.60 for comparison of change in liver fat by remission status, p = 0.029 for comparison of achieved liver fat by remission status).

Adverse events reported during the intervention included diarrhea (n = 1 (4%)), constipation (n = 9 (39%)), dizziness (n = 1 (4%)), headache (n = 9 (39%)), mood change (n = 1 (4%)), hair loss (n = 3 (13%)), fatigue (n = 1 (4%)) and other (n = 1 (4%)). Self-reported severity was generally mild or moderate, with one participant reporting severe constipation and diarrhea at different visits (Supplementary Table S4).

**Fig. 3:**
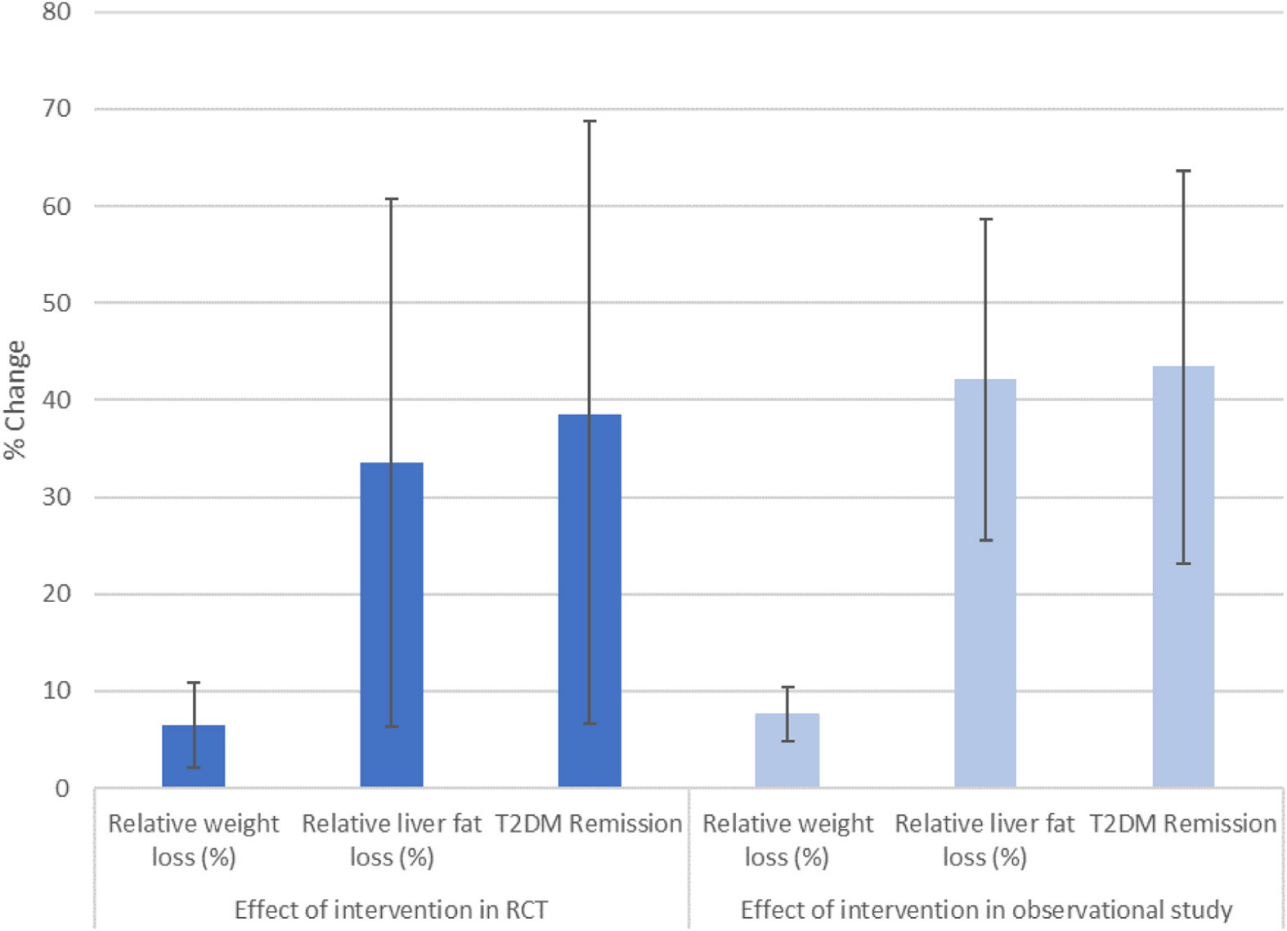
Relative weight loss, relative liver fat loss, and relative change in remission in the RCT phase (n = 25, ITT: intervention compared to control) and in the observational phase (n = 23, ITT: change following TDR).

**Fig. 4:**
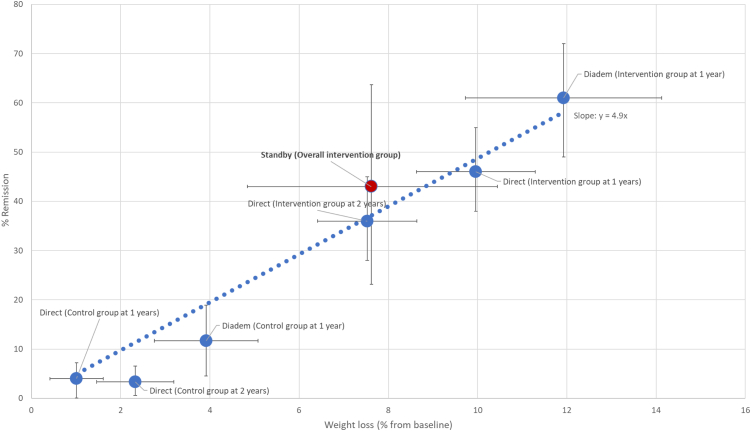
Relationship between relative weight loss and achieving remission in STANDby, DIRECT 1 and 2 year follow-up studies, and DIADEM-I. Bars are 95% CI.

Fig. 3 summarises relative changes in weight, liver fat, and remissions in both the RCT and observational parts of the study, showing broadly concurrent results. Fig. 4 shows the percentage achieving remission by relative weight loss in the STANDBY, DIRECT, and DIADEM-I trials. STANDBY achieved results broadly in line with results from these published trials.

Weight loss was also associated with changes in other measurements, including sytolic blood pressure, insulin and HOMA-IR, as well as change in triglycerides, C-reactive protein, and Eq5D (Table 4).

**Table 4:** Association of change in variables of interest (exposure) with change in weight (outcome in Kg) in univariable linear regression (n = 23, ITT analysis).

Variable	Beta coefficient	Lower 95% CI	Upper 95% CI	p-value	Standardised beta^a^
HbA1c (per mmol/mol)	0.24	−0.07	0.56	0.12	0.455
Waist circumference (per cm)	0.36	−0.03	0.74	0.07	0.336
SBP (per mmHg)	0.18	0.05	0.32	0.009	0.465
Glucose (per mmol/L)	1.39	−0.11	2.90	0.07	0.427
Total cholesterol (per mmol/L)	0.07	−2.54	2.67	0.96	0.012
HDL-cholesterol (per 0.1 mmol/L)	0.67	−0.37	1.71	0.19	0.237
Triglycerides (mmol/L)	1.69	0.70	2·68	0.002	0.292
Urea (mmol/L)	−1.47	−5.02	2.08	0.40	−0.256
Uric acid (mg/dL)	−1.89	−5.54	1.75	0.29	−0.315
Estimated glomerular filtration rate (ml/min/1.73 m^2^)	0.18	−0.20	0.56	0.34	0.277
GGT (per U/L)	0.02	−0.09	0.13	0.68	0.097
ALT (per U/L)	−0.13	−0.56	0.30	0.53	−0.230
AST (per U/L)	−0.34	−0.96	0.27	0.26	−0.370
C-reactive protein (per mg/L)	1·02	0.71	1.34	<0.001	0.694
Ferritin (per ug/L)	−0.03	−0.16	0.11	0.70	−0.112
Insulin (per uU/ml)	0.43	0.33	0.53	<0.001	0.846
HOMA-IR (per unit)	1.17	0.79	1.55	<0.001	0.835
Total adipose tissue volume (per L)	1.53	1.34	1.72	<0.001	0.923
Abdominal subcutaneous adipose tissue (L)	3.46	2.96	3.96	<0.001	0.894
Lean volume (per L)	4.11	0.67	7.54	0.02	0.599
Liver fat (per 1%)	0.01	0.00	0.01	0.007	0.647
Visceral adipose tissue volume (L)	6.07	2.80	9.35	<0.001	0.759
EQ5D (VAS units)	−0.11	−0.21	0.00	0.045	−0.358

A positive beta coefficient indicates that as the variable of interest decreases, weight decreases.

a The beta coefficient between standardised change in the exposure of interest with standardised change in BMI (i.e. allowing subjective comparison of strength of association of change in different exposures with change in BMI).

### Programme acceptability to recruited volunteers

As well as demonstrating acceptability by achieving weight loss for most of the participants (insofar as weight loss shows people must have been taking the low-calorie diets), acceptability of the programme content was assessed by written (or verbal in a small number of cases) answers to three open-ended questions administered by the research dietitian on the final study appointment. Of 23 participants who started the programme, 16 answered at least one question. In response to the question “*What did you like about Counterweight Plus?*” nine participants mentioned weight loss, feeling good on the diet, feeling good with calorie reduction, or meeting their weight target. Five participants mentioned dietitian support or health checks, and eleven mentioned liking the flavour or ease of the diet. Asked “*What did you not like about Counterweight Plus*”, eight disliked some, or most, of the flavours, two mentioned gastrointestinal side effects, one mentioned hair loss. Asked “*Anything you would suggest to improve Counterweight Plus for other patients in future?*”, three said they liked the diet and suggested no improvements, six mentioned different varieties of meal replacements or extending meal replacement duration, and three suggested using exclusively or mixed food-based approaches instead of TDR (Further details in Supplementary Table S5). No serious adverse events were reported, though some did report constipation or headaches.

## Discussion

This proof-of-concept study was designed to answer important unanswered research questions about T2D management in South Asians, who make up around a quarter of the entire world's population. The hypothesis, that the mechanisms of T2D and its remission are similar to those in white Europeans, has not yet been demonstrated in a randomised trial, thus weight management has not yet been confidently proposed for South Asians for this purpose. The present study was designed to establish, as proof of principle, whether weight loss would lead to remissions of T2D in South Asians, and to try to assess applicability of this type of management to South Asian people. Our results, accumulated in part during the pandemic with all its inevitable obstacles, indicate that, despite well-known differences in disease aggressiveness, the role of excess adiposity, particularly ectopic liver fat in the liver, in T2D and its reversal to achieve remission are broadly the same, and that similar treatment approaches should in principle be appropriate for South Asian people with T2D.

Assuming similarly very large effect sizes to those in DiRECT and DIADEM-I, a total study size of n = 20 was sufficient to prove intervention efficacy to generate weight loss >2.8 kg in the randomised trial part, and greater study power was provided for the observational study for remission of diabetes. The study design also provided an incentive for retention of participants allocated to the control group, which is problematic for a highly valued dietary intervention which is necessarily unblinded. Because ethical committees have not yet accepted use of telephone, text or email to recruit research participants, only conventional postal services were used for personal invitations. Using this approach, as in the DiRECT trial, recruitment methods were relatively low in South Asians at 12%. The initial uptake in DIRECT was similar, but after a substantial protocol amendment recruitment was supplemented by phone calls, to those who did not respond to postal invitations and reminders, reached 28%, a high figure for primary care recruitment. Self-referral from media advertisements is a much more efficient method, probably attracting more interested or motivated individuals. Acceptability in those recruited was indicated by retention of 19/23 (83%) of participants who started TDR, and the overall responses to questionnaires were positive (Supplementary Table S3). However, the strongest objective indicator of acceptability and adherence for any lifestyle-based (i.e., behavioral) weight management programme is weight loss, as programme adherence will always lead necessarily to weight change, thus effectiveness. The 7.6% average weight loss was less than was observed by white UK participants in DiRECT, in part as the calorie deficit was less in the lighter South Asians. Approximately a third achieved under 5% weight loss, indicating only brief engagement with the TDR and so this proportion found the intervention minimally acceptable. The somewhat lesser weight losses by South Asians observed in STANDby are in line with early data from the NHS England Diabetes Prevention Program^16^ and the NHS England type 2 diabetes remission pilot.^17^ There is clearly an opportunity to tailor and refine the intervention, and better present the opportunity to achieve T2D remission, for South Asians, but with the existing Counterweight-Plus programme about a third lost over 10% weight, and over 40% of all who commenced the programme achieved remissions. The mean weight loss necessary to achieve T2D remission was about 8 kg. These observations are restricted to the volunteers, mostly English-speaking Pakistani ancestry living in Scotland, who are not necessarily representative of the wider South Asian population, for whom broader assessments of intervention acceptability will be needed. Responses to questions about the programme, which might inform improvements, mainly focused on formula diet flavours, and no serious obstacles were identified.

The RCT results confirmed that the intervention generated more remissions than standard care. Both RCT and the enlarged observational cohort demonstrated about 40% remissions in all those who started TDR, close to the 46% at 12 months in DiRECT. The DIADEM-I trial^11^ achieved even a higher remission rate, 61% at 12 months with greater weight loss in a population with shorter duration diabetes. Plotted together with results from DIRECT after one and two years,^8^^,^^18^ and DIADEM-I, the present STANDBY results fall in line with expected remission rate for *percent* weight loss (Fig. 4), in line with the concept that weight loss rapidly improves glycaemia (and that the degree of weight loss aligns tightly to the chances of remission) in keeping with T2D as a disease of ectopic fat. Quality of life improved significantly in the overall group after intervention, and by about the same magnitude as in DIRECT.

The participants had a mean baseline BMI of 32 kg/m^2^, lower than in DiRECT (34.6 kg/m^2^) or DIADEM-I (34.4 kg/m^2^ male, 36.2 kg/m^2^ female), and substantially lower mean body weight at 88.2 kg, reflecting their shorter statures. Lesser absolute weight loss in STANDby than in other trials could be partially explained by the intervention starting with the same formula total diet replacement, ∼850 kcal/day, thus a smaller energy deficit for smaller, relatively inactive, participants whose metabolic rates will have been proportionally lower. Even so, there appears room to improve some aspects of the intervention to improve and widen its acceptability to more South Asians.

The DiRECT trial confirmed earlier suggestions from Counterpoint^19^ that ectopic fat, specifically liver fat likely plays a key role in the pathogenesis of T2D and its remission, in line with recent genetic data.^20^ We have now shown similarly high liver fat contents at baseline (mean 15.6% vs 16.0% in DIRECT),^9^ measured by MRI, in South Asians with T2D, despite their lower weight. These data fit with recent evidence showing South Asians without diabetes carry more liver fat at similar BMIs to their white counterparts.^21^ As in DiRECT, remission was closely related to reduction in excess liver fat. The liver fat reduction was a little more modest in STANDby than in DIRECT,^22^ again in keeping with lesser weight loss. Both these fat mass changes reflect a general loss of intra-abdominal ectopic fat deposits, and liver fat appears to be causally related to T2D.^20^ The robust reduction in fasting insulin concentration, and HOMA, are in keeping with improved tissue insulin sensitivity in tissues such as the liver. It thus appears that the mechanism of T2D and its remission may indeed be broadly the same in South Asians as in Europeans.

Whilst the present study has demonstrated how a carefully designed, highly economic, study with modest but sufficient numbers, can provide meaningful outcome data, it does have several important limitations. The study was severely challenged by its timing, which coincided with the COVID-19 pandemic, necessitating some design changes, some post hoc, to attempt to overcome obstacles to obtain concurrent body weight, MRI and HbA1c results. With these design amendments and some missing data, we had to limit analyses to slightly shorter-term outcomes than originally intended, so longer sustainability of weight loss and remission in South Asians needs future testing. The findings in mostly English-speaking Pakistani living in Scotland are encouraging, and consistent with wider literature, but cannot be considered definitive for all people of South Asian origin with type 2 diabetes, given the trial was only powered to prove remission for the iTDR and dTDR phases combined, versus the control remission rate (albeit high at 12%) from DIADEM-I. Nevertheless, remissions were significantly reduced in both the RCT and combined phases, so despite some differences in baseline characteristics between the iTDR and dTDR groups, remission data appear likely to be robust. We accept that the patients recruited were likely more highly motivated, given barriers during COVID, and recognise that South Asians tend less often to volunteer for medical studies. Further future work will need to explore ways to broaden programme acceptability, with weight change as the principle measurable outcome. We set an intervention target for individuals of 15 kg weight loss, as in DiRECT, in an effort to maximise the numbers who would achieve >10 kg loss: whether this is an optimal practical strategy needs to be explored. Finally, most of the patients were of Pakistani origin, but as all ethnically diverse South Asian groups are at much greater risk of type 2 diabetes than are white people, our results should be of interest to South Asians in general.

The STANDby study design, adding a delayed intervention control group, was effective in retaining control group participants and in augmenting numbers for observational evaluations. This can provide a useful model for future trials in other population groups of a desired intervention with a large effect size. The relatively small number recruited does allow questions about representativeness of the sample, but it was more ethical given prior results and the rapid enthusiastic response to an advertisement on social media suggests that the intervention is attractive to large numbers. Longer-term outcome data will be valuable for clinical guidelines, but the principle of remission achievement has been demonstrated, and can act as a strong spur to other South Asian communities and countries, particularly if the seriousness of the diagnosis is better understood. The mechanisms behind T2D remission in the STANDby sample appear to be the same as in DiRECT, but there may yet be variations across the massive global populations of South Asians with diabetes. Although attractive and broadly acceptable to volunteer participants from the Scottish South Asian population, substantial cultural and ethnic variations exist across the diverse global population, so well-researched and tailored interventions will be needed to optimise the numbers who can achieve and benefit from remission of type 2 diabetes. A wide range of dietary options including TDR, meal replacements, and food-based diet programmes that are tailored to South Asians are urgently needed.

A TDR-led structured weight management programme is attractive and appears acceptable to UK-based South Asians who agree to undertake the intervention. There is, however, room to improve outcomes, but one-third lost >10% body weight and the mechanism underpinning a 43% diabetes remission rate appears the same as in white Europeans, involving loss of excess ectopic body-fat and resultant improved insulin sensitivity. These results provide important proof of concept for a disabling disease that is rising rapidly in an ethnic group that makes up a quarter of the world's population. Further larger, longer, pragmatic studies are now needed to gauge and refine acceptability and sustainability of this approach in South Asians living in different parts of the world.

## Contributors

NS and MEJL conceived the idea for this trial. All authors contributed to the study design and conduct. NS, WL, PW and MEJL wrote the paper and all authors helped revise it critically for important intellectual content.

## Data sharing statement

Data can be made available upon reasonable request to corresponding authors after key papers have been published. The full trial protocol may be obtained on reasonable request from the corresponding author.

## Declaration of interests

PW reports grant income from Roche Diagnostics, Astrazeneca, Boehringer Ingelheim, and Novartis, and speaker fees from Novo Nordisk, outside the submitted work. NS has received grant and personal fees from AstraZeneca, Boehringer Ingelheim, and Novartis; grant from Roche Diagnostics; and personal fees from Abbott Laboratories, Afimmune, Amgen, Eli Lilly, Hanmi Pharmaceuticals, Janssen, Merck Sharp & Dohme, Novartis, Novo Nordisk, Pfizer, and Sanofi outside the submitted work. ML declares department/university grant funding from NIHR, All Saints Educational Trust and Diabetes UK, and personal fees for lecturing and advisory boards from Novo Nordisk, Roche, Merck, Sanofi, Nestle and Oviva. All other authors declare no conflicts of interest.

## Appendix A Supplementary data

STANDby Supplementary Material

STANDby Protocol
