# Supplementary material for: Dietary weight-management for type 2 diabetes remissions in South Asians: the South Asian diabetes remission randomised trial for proof-of-concept and feasibility (STANDby)
Source: Lancet Reg Health Southeast Asia. 2022 Nov 23;9:100111. doi: 10.1016/j.lansea.2022.100111 (PMC9904218; doi:10.1016/j.lansea.2022.100111)
Supplement: STANDby Supplementary Material [file mmc1.pdf]

SUPPLEMENT

Dietary weight-management for type 2 diabetes remissions in South Asians: The South Asian diabetes remission randomised trial for proof-of-concept and feasibility (STANDby)

Naveed Sattar, Paul Welsh, Wilma S Leslie, George Thom, Louise McCombie, Naomi Brosnahan, Janice Richardson, Jason MR Gill, Luisa Crawford, Michael EJ Lean

Supplementary Table 1 Change in outcome variables during the RCT (PP analysis)

Factor	Change in dTDR (control)	Change in iTDR	p-value	Between group change (95% CI)
	N=12	N=13		
Weight (Kg)	-1.1 (1.3) (n=10)	-7.8 (7.8) (n=12)	0.015	-6.7 (-11.9, -1.5)
Weight (% change)	-1.4 (1.5) (n=10)	-8.3 (7.1) (n=12)	0.007	-6.9 (-11.7, -2.1)
Weight change category				
<5% weight loss	10 (100%)	4 (33%)	0.004	-
5-9.9% weight loss	0 (0%)	2 (17%)		-
>10% weight loss	0 (0%)	6 (50%)		-
Remission	0 (0%)	5 (38%)	0.039	-
HbA1C (mmol/mol)	-0.9 (6.2) (n=10)	-6.5 (11.7) (n=11)	0.20	-5.6 (-14.3, 3.2)
Taking antidiabetic medication	7 (70%) (n=10)	2 (17%) (n=12)	0.027	-
Taking antihypertensive medication	4 (50%) (n=8)	0 (0%) (n=9)	0.029	-
Waist circumference (cm)	0.2 (5.3) (n=10)	-6.2 (5.7) (n=10)	0.017	-6.5 (-11.6, -1.3)
SBP (mmHg)	-6.2 (19.1) (n=10)	-4.3 (11.5) (n=12)	0.77	1.9 (-11.8, 15.6)
Glucose (mmol/L)	-0.6 (1.8) (n=10)	-1.2 (1.6) (n=11)	0.41	-0.6 (-2.2, 0.9)
Total cholesterol (mmol/L)	-0.1 (0.4) (n=10)	-0.1 (1.2) (n=11)	0.92	-0.0 (-0.9, 0.8)
HDL-cholesterol (mmol/L)	0.0 (0.2) (n=10)	0.0 (0.2) (n=11)	0.83	0.0 (-0.1, 0.2)
Triglycerides (mmol/L)	-0.3 (0.8) (n=10)	0.0 (1.5) (n=11)	0.59	0.3 (-0.8, 1.4)
Urea (mmol/L)	0.1 (1.3) (n=10)	0.2 (1.2) (n=11)	0.83	0.1 (-1.0, 1.2)
Uric acid (mg/dL)	-0.3 (0.7) (n=10)	0.1 (1.4) (n=11)	0.34	0.5 (-0.5, 1.4)
Estimated glomerular filtration rate	1.3 (6.5) (n=10)	-0.6 (11.4) (n=11)	0.65	-1.9 (-10.5, 6.7)
GGT (U/L)	-20 (73) (n=10)	-3 (16) (n=11)	0.47	17 (-31, 64)
ALT (U/L)	-3 (8) (n=10)	-3 (12) (n=11)	0.98	0 (-10, 10)
AST (U/L)	-2 (4) (n=10)	1 (8) (n=11)	0.32	3 (-3, 8)
C-reactive protein (mg/L)	-1.0 (2.4) (n=10)	-3.2 (5.9) (n=11)	0.27	-2.3 (-6.5, 1.9)
Ferritin (ug/L)	-9 (18) (n=10)	6 (37) (n=11)	0.26	15 (-12, 42)
Insulin (uU/ml)	-2.8 (8.8) (n=10)	-9.4 (16.3) (n=11)	0.27	-6.6 (-18.7, 5.6)
HOMA-IR	-2.2 (4.9) (n=10)	-3.8 (5.5) (n=11)	0.48	-1.6 (-6.4, 3.2)
Total adipose tissue volume (L)	-0.7 (0.8) (n=9)	-5.1 (4.5) (n=11)	0.009	-4.4 (-7.6, -1.3)
Abdominal subcutaneous adipose tissue (L)	-0.2 (0.5) (n=9)	-1.9 (2.0) (n=11)	0.021	-1.8 (-3.2, -0.3)
Lean volume (L)	-0.1 (0.4) (n=9)	-0.9 (1.1) (n=11)	0.036	-0.8 (-1.6, -0.1)
Liver fat (%)	-1.8 (3.5) (n=9)	-8.2 (8.2) (n=11)	0.044	-6.4 (-12.6, -0.2)
Visceral adipose tissue volume (L)	-0.1 (0.4) (n=9)	-1.1 (0.7) (n=11)	0.001	-1.0 (-1.5, - 0.5)

Data are for within group change are count (%) or mean (SD)

Supplementary Table 2 Adverse events during the RCT phase

Adverse Event	dTDR (control)	iTDR	p-value
	N=12	N=13	
Diarrhea	0 (0%)	0 (0%)	0.99
Nausea	0 (0%)	0 (0%)	0.99
Constipation	0 (0%)	3 (23%)	0.22
Dizziness	0 (0%)	1 (8%)	0.99
Indigestion	0 (0%)	0 (0%)	0.99
Headache	0 (0%)	4 (31%)	0.096
Mood change	0 (0%)	1 (8%)	0.99
Cold sensitivity	0 (0%)	0 (0%)	0.99
Hair loss	0 (0%)	2 (15%)	0.48
Fatigue	0 (0%)	1 (8%)	0.99
Other	0 (0%)	1 (8%)	0.99

Supplementary Table 3 Change in outcome variables from baseline, after intervention in all participants (combined iTDR and dTDR) (PP analysis)

Factor		Change	Lower 95% CI	Upper 95%CI	p-value
Weight (Kg)	20	-7.8	-10.7	-4.9	<0.001
Weight (% change)	20	-8.8	-11.6	-5.9	<0.001
Weight change category n (%)					<0.001
<5% weight loss	6 (30%)	-	-	-	
5-9.9% weight loss	6 (30%)	-	-	-	
>10% weight loss	8 (40%)	-	-	-	
Remission (%)	21	10 (48%)	26%	69%	<0.001
HbA1C (mmol/mol)	21	-7.9	-13.5	-2.3	0.008
Change in taking antidiabetic medications	20	-6 (-30%)	-59%	0%	0.07
Change in taking antihypertensive medication	17	0 (0%)	-22%	22%	0.99
Waist circumference (cm)	18	-6.5	-9.5	-3.5	<0.001
SBP (mmHg)	20	-8.3	-15.1	-1.5	0.019
Glucose (mmol/L)	19	-1.4	-2.5	-0.4	0.007
Total cholesterol (mmol/L)	19	-0.3	-0.9	0.2	0.24
HDL-cholesterol (mmol/L)	19	-0.1	-0.2	0.1	0.30
Triglycerides (mmol/L)	19	-0.1	-0.7	0.5	0.65
Urea (mmol/L)	19	0.1	-0.5	0.7	0.65
Uric acid (mg/dL)	19	0.2	-0.4	0.8	0.46
Estimated glomerular filtration rate (ml/min/1.73 m^2)	19	-0.5	-5.9	4.8	0.83
GGT (U/L)	19	-12.5	-27.2	2.2	0.09
ALT (U/L)	19	-4.2	-10.1	1.8	0.16
AST (U/L)	19	-0.3	-3.9	3.4	0.88
C-reactive protein (mg/L)	19	-2.4	-4.7	-0.2	0.04
Ferritin (ug/L)	19	2.4	-12.8	17.6	0.75
Insulin (uU/ml)	19	-7.7	-14.3	-1.2	0.02
HOMA-IR	19	-3.4	-5.8	-1.1	<0.001
Total adipose tissue volume (L)	18	-4.9	-6.8	-3.1	<0.001
Lean volume (L)	18	-0.6	-1.1	-0.1	0.02
Liver fat (%)	18	-8.6	-12.0	-5.2	<0.001
Visceral adipose tissue volume (L)	18	-1.3	-1.6	-0.9	<0.001
EQ5D (VAS units)	21	12.2	2.1	22.4	0.02

Supplementary Table 4 Adverse events during the intervention in all participants (combined iTDR and dTDR)

Adverse Event	All participants
	N=23
Diarrhea	1 (4%)
Nausea	0 (0%)
Constipation	9 (39%)
Dizziness	1 (4%)
Indigestion	0 (0%)
Headache	9 (39%)
Mood change	1 (4%)
Cold sensitivity	0 (0%)
Hair loss	3 (13%)
Fatigue	1 (4%)
Other	1 (4%)

Supplementary Table 5 Acceptability questionnaire: Curated responses to open questions regarding the diet (rows are not matched responses for a single participant). N=16 participants responded to at least one question.

What did you like about Counterweight Plus?	What did you not like about Counterweight Plus?	Anything you would suggest to improve Counterweight Plus for other patients in future?
Felt good, helped change my lifestyle, liked all flavours.	Disliked some flavours	I would recommend the diet to others
Helped me lose weight, liked the flavours, didn't make me feel hungry	Disliked many flavours/meals. Customized own diet/exercise plan around intervention	Stick to food based approaches
Helped me lose weight and gave me motivation. Exercised a lot. Health checks welcome.	Nothing to dislike	More flavours, spicy too, less with milk
Liked the reduced calories. Like the food products. Health checks welcome.	Wind and bloating particularly in the first week	Mixing TDR with some solid food
Liked structure for regimented in calorie intake. The products were varied and not distasteful.	Diarrhea	More flavours in the soups and shakes. Longer duration of TDR
Liked dietitian support during the intervention. The soups and shakes were flavoursome.	Dietitian was changed - gap in continuity	Brilliant diet, don't think improvements needed
Liked weight loss - encouraging and motivating.	Hair loss, boring flavours	Not anything I can think of now
Liked weight loss. Diabetes better.	Miss food, use the shakes selectively	More spicy options. Food based approach
Quick and easy to use meals.	Disliked many flavours/meals, & not filling	Resolve side-effects
Liked feedback, encouragement, and support.	Disliked some flavours (too sweet)	More variety
Liked specific flavours. Tastes close to normal food.	Boring diet	Full variety of listed meal replacements in promotional material should be available
Tasty and easy. Reassuring to know you are getting required vitamins.	Disliked many flavours/meals (too sweet, too salty). Overall, not enjoyable to do.	Support should carry on a bit longer
Liked specific flavours.	Difficult in adherence especially in lockdown. Only managed 2 shakes per day with one meal.	More emphasis for all with diabetes to lose weight to reduce or avoid the need for medication
Really easy to use and worked around my lifestyle. I was able to achieve my desired goals	Nothing to dislike	Extra meal replacements at completion of TDR
Liked weight loss. Liked feedback, encouragement, and support.		
