# Supplementary material for: Dietary weight-management for type 2 diabetes remissions in South Asians: the South Asian diabetes remission randomised trial for proof-of-concept and feasibility (STANDby)
Source: Lancet Reg Health Southeast Asia. 2022 Nov 23;9:100111. doi: 10.1016/j.lansea.2022.100111 (PMC9904218; doi:10.1016/j.lansea.2022.100111)
Supplement: STANDby Protocol [file mmc2.pdf]

Sou**T**h **A**sia**N** **D**iabetes remission feasi**B**ility stud**Y**

(STANDby)

**Running title:**	**STANDby**
**Protocol Version:**	**12**
**Date:**	**28.01.2022**
**REC Reference Number:**	**17/WS/0104**
**ISRCTN/Clinical trial.gov:**	http://www.isrctn.com/ISRCTN10720065
**Sponsor's Protocol Number:**	**GN16DI1505**
**Sponsor:**	**NHS Greater Glasgow & Clyde**
**Funder:**	**Department of Human Nutrition, University of Glasgow**<br>**Department of Metabolic Medicine, University of Glasgow**

Amendment number	Date	Protocol version
1	January 2018	2
2	September 2018	3
(Minor Amendment)	February 2019	4
3	July 2019	5
4	23rd July 2019	6
5	17th December 2019	7
5 (revised)	31st January 2020	8
6	25th March 2020	9
7	08.09.2020	10
8	07.10.2020	11
9	28.012021	12

This study will be performed according to the Research Governance Framework for Health and Community Care (Second edition, 2006) and WORLD MEDICAL ASSOCIATION DECLARATION OF HELSINKI Ethical Principles for Medical Research Involving Human Subjects 1964 (as amended).

CONTACTS

Chief Investigators

Professor Michael EJ Lean
Professor of Human Nutrition
Department of Human Nutrition, Glasgow Royal Infirmary
10-16 Alexandra Parade, Glasgow, G31 2ER
Tel: 0141-211-4686
E-mail: Mike.Lean@glasgow.ac.uk

Professor Naveed Sattar
Professor of Metabolic Medicine
RC214 Level C2, Institute of Cardiovascular and Medical Sciences, BHF GCRC
Tel: 0141-330-3419
E-mail: Naveed.Sattar@glasgow.ac.uk

Co-investigators

Naomi Brosnahan
Research Associate, DIRECT Study
Human Nutrition, School of Medicine - GRI Campus
College of Medical, Veterinary and Life Sciences University of Glasgow,
Room 2.21, 2nd Floor, New Lister Building Glasgow Royal Infirmary
10-16 Alexandra Parade, Glasgow G31 2ER
Tel: 07701-281-651
E-mail: naomi.brosnahan@glasgow.ac.uk

Dr Jason Gill
BHF Glasgow Cardiovascular Research Centre
Institute of Cardiovascular and Medical Sciences
College of Medical, Veterinary and Life Sciences
University of Glasgow, Glasgow, G12 8TA
Tel: 0141 3302916
Email: Jason.Gill@glasgow.ac.uk

Dr Wilma S Leslie
Study Coordinator, DiRECT study
Human Nutrition, School of Medicine - GRI Campus
College of Medical, Veterinary and Life Sciences University of Glasgow,
Room 2.21, 2nd Floor, New Lister Building Glasgow Royal Infirmary
10-16 Alexandra Parade, Glasgow, G31 2ER
Tel: 0141-201-8609
Email: Wilma.Leslie@glasgow.ac.uk

Louise McCombie
Research Associate, DIRECT Study
Human Nutrition, School of Medicine - GRI Campus
College of Medical, Veterinary and Life Sciences University of Glasgow,
Room 2.21, 2nd Floor, New Lister Building Glasgow Royal Infirmary
10-16 Alexandra Parade, Glasgow G31 2ER
Tel: 07968-820-081
E-mail: louise.mccombie@glasgow.ac.uk

Janice Richardson
Research Nurse
Institute of Cardiovascular and Medical Sciences, BHF GCRC, Glasgow, G12 8TA,
Tel: 0141-3307-087
E-mail**:** Janice.richardson@glasgow.ac.uk

Anna Bell-Higgs
Counterweight Ltd.
Stafford House, 10 Brakey Road, Corby, Northants, NN17 5LU
Tel: 07701281653
Email: anna.bell-higgs@counterweight.org

George Thom
Research Associate, DIRECT Study
Human Nutrition, School of Medicine - GRI Campus
College of Medical, Veterinary and Life Sciences University of Glasgow,
Room 2.21, 2nd Floor, New Lister Building Glasgow Royal Infirmary
10-16 Alexandra Parade, Glasgow G31 2ER
Tel: 07515-552-173
E-mail: george.thom@glasgow.ac.uk

Collaborators:
Dr Dalia Malkova
Senior Lecturer
Human Nutrition, School of Medicine, - GRI Campus
College of Medical, Veterinary and Life Sciences University of Glasgow,
New Lister Building Glasgow Royal Infirmary
10-16 Alexandra Parade, Glasgow, G31 2ER
E-mail: Dalia.Malkova@glasgow.ac.uk

Dr Nazim Ghouri
Consultant Diabetologist, Queen Elizabeth University Hospital, & Honorary Clinical Senior Lecturer
Institute of Cardiovascular and Medical Sciences
Leel C2, Institute of C&MS, BHF Gcrc
Glasgow G12 8TA
Nazim.Ghouri@glasgow.ac.uk

Dr Giles Roditi
Consultant Cardiovascular Radiologist, NHS Greater Glasgow & Clyde & Honorary Clinical Associate Professor, University of Glasgow
School of Medicine
University of Glasgow
Giles.Roditi@glasgow.ac.uk

Sponsor - NHS Greater Glasgow and Clyde

R&D Representative – NHS Greater Glasgow and Clyde
Dr Maureen Travers,
Research Co-ordinator, NHS Greater Glasgow & Clyde
Clinical Research & Development
West Glasgow Ambulatory Care Hospital
Dalnair Street, Glasgow, G3 8SW
Tel: +44(0) 141 232 1813
E-mail: Maureen.Travers@ggc.scot.nhs.uk

Funding Body

Department of Human Nutrition, University of Glasgow
Department of Metabolic Medicine, University of Glasgow

PROTOCOL APPROVAL

South **A**sia**n** **D**iabetes Remission Feasibility Study (STANDby)

Chief Investigators

Professor Michael EJ Lean
Professor of Human Nutrition
Department of Human Nutrition, Glasgow Royal Infirmary
10-16 Alexandra Parade, Glasgow, G31 2ER, Glasgow

Signature:

Date:25.04.2017

Professor Naveed Sattar
Professor of Metabolic Medicine
RC214 Level C2, Institute of Cardiovascular and Medical Sciences, BHF GCRC

Signature:

Date:25.04.2017

Sponsor's representative

Dr Maureen Travers
Research Co-ordinator
NHS Greater Glasgow & Clyde
Research and Development Management Office
Tennent Institute
38 Church Street
Western Infirmary
Glasgow G11 6NT

Signature:

Date:

TABLE OF CONTENTS

Once protocol draft complete, left click on this section press F9 and update entire table

CONTACTS 2
PROTOCOL APPROVAL 4
TABLE OF CONTENTS 5
ABBREVIATIONS 6
STUDY SYNOPSIS 7
STUDY FLOW CHART 11
1. INTRODUCTION 12
1.1 Background: the problem 12
1.2 Rationale 12
1.3 Prior experience of managing obesity and type 2 diabetes 13
1.4 Study hypothesis 13
2. STUDY OBJECTIVES 13
Research Questions 13
3. STUDY DESIGN 13
3.1 Study Population 14
3.2 Inclusion criteria 14
3.3 Exclusion criteria 14
3.4 Identification of participants and consent 15
3.4.1 Non respondents 15
3.5 Withdrawal of subjects 16
4 Trial procedures 16
4.1 Study schedule (App A) 18
4.2 Study Outcome Measures 20
4.3 Laboratory Tests 20
5. ASSESSMENT OF SAFETY 21
6.1 Sample size 22
6.2 Management and delivery 22
7.0 STUDY CLOSURE / DEFINITION OF END OF STUDY 22
8. Data Handling 22
8.1 Case Report Forms / Electronic Data Record 22
8.2 Record Retention 22
9. STUDY MONITORING/AUDITING 23
10. PROTOCOL AMENDMENTS 23
11. ETHICAL CONSIDERATIONS 23
12. INSURANCE AND INDEMNITY 23
13. FUNDING 23
14. ANNUAL REPORTS 23
APPENDIX A: STANDby SCHEDULE OF ASSESSMENTS 25
APPENDIX B: Details of Mechanistic Studies Error! Bookmark not defined.
APPENDIX C: Sample Handling Manual 26
Appendix D: Counterweight Plus Programme Appointment Planner 27

ABBREVIATIONS

App	Appendix
Appt	Appointment
BMI	Body Mass Index
CHD	Coronary Heart Disease
CW	Counterweight
eGFR	Estimated Glomerular Filtration Rate
ITT	Intention to Treat
LELD	Low Energy Liquid Diet
MR	Magnetic Resonance Imaging
NICE	National Institute for Health and Clinical Excellence
OHA	Oral Hypoglycaemic Agent
RCT	Randomised Controlled Trial
SIGN	Scottish Intercollegiate Guideline Network
T2DM	Type 2 Diabetes Mellitus
TDR	Total Diet Replacement
VLDL	Very Low Density Lipoprotein
MR	Metabolic Rate

STUDY SYNOPSIS

Title of Study:	South Asian Diabetes Remission Feasibility Study (STANDby)
Study Centre:	University of Glasgow
Duration of Study:	1 year
Primary Objective:	To determine the feasibility and acceptability of an intensive weight management programme in South Asian patients to achieve remission of diabetes at 3 months.
Secondary Objective:	To assess weight change, HBA1c, quality of life, process evaluations, mechanistic changes to inform a larger randomised controlled trial
Primary Endpoint:	Percentage achieving ≥15kg weight loss, and/or HbA1c <48mmol/l, off treatment, at end Total Diet Replacement stage to inform larger randomised controlled trial
Rationale:	This proposal is for a small pilot study in South Asian patients,, essentially identical to the protocol used by the same research team in the Type 2 diabetes Remission Clinical Trial (DiRECT) study in Caucasian patients, which has now completed recruitment of 290 patients. Published research in people of European origin demonstrates that low/very low energy diets achieving around 15 kg weight loss usually lead to diabetes remission, after 8 weeks of treatment. The Diabetes Remission Clinical Trial (DiRECT) is currently underway in Tyneside and Scotland to assess long-term outcomes of this treatment approach in primary care. However, to date research has not been conducted using this approach with patients of South Asian origin. South Asians are typically diagnosed with Type 2 diabetes 5-10 years younger, with a briefer period of 'prediabetes', and at a lower body mass index than white Europeans. For any given BMI, South Asians have higher fat mass and a larger proportion of fat mass in deep intra-abdominal visceral depots and in ectopic sites: there is emerging evidence for greater liver and pancreas fat, driving type 2 diabetes. This is potentially reversible, but conventional weight management methods commonly achieve less weight loss among South Asians. The proposed study will assess if an existing evidence-based weight management programme, 'Counterweight-Plus', is acceptable to patients of South Asian origin and achieves weight change and reversal of T2DM compared with routine care. The Primary focus being acceptability of TDR in this population and response in terms of weight change and remission of diabetes following the TDR. Initial findings will be used to inform a larger randomised controlled trial.
Methodology:	**The study design** for this pilot will be a randomized controlled trial. Participants will be randomised to either commence the intervention immediately, or continue current treatment for T2DM for the 3 months, prior to receiving the intervention. After this period of controlled observation, those allocated to the control arm will be offered the intervention, in order to increase the numbers of patients providing information on acceptability and to gauge the likely distribution of weight changes. This information will be used for a future power calculation.

	At baseline and again after weight loss has been completed and weight stabilized (16-20 weeks), all participants will complete questionnaires on Quality of Life and have blood samples and anthropometric data collected. A questionnaire exploring participant's weight loss expectations and current diet will be administered at baseline.<br>Treatment acceptability will be assessed by questionnaire at the end of the study period<br>**Intervention arm**, consenting patients will undertake Counterweight-Plus, delivered by a research dietitian. Any existing anti-diabetic, anti-obesity and antihypertensive medications will be withdrawn at the start of 825-853kcal/day 'Total Diet Replacement' liquid diet phase. If necessary they will be reintroduced using standardised protocols according to blood glucose and BP increases. All other management of type 2 diabetes will continue as per clinical guidelines.<br>A questionnaire to quantify psychological process measures (motivation, self-efficacy, resources, self-regulation, habit, satisfaction with outcomes, diet/behaviour, goal conflict, goal facilitation, social support and environmental support) will be completed by participants at each intervention visit<br><br>An MRI scan will be performed at baseline and repeated after weight loss.<br><br>Participants will be invited to undergo additional measurements of Metabolic Rate (resting and during physical activity) and Body Composition. These tests will be optional<br><br>**Control arm,** patients will continue to be treated according to clinical guidelines.
Sample Size:	25
Screening:	Potential participants will be identified by a computerised search of patient records in participating GP practices. A research nurse will conduct this. Searches will be undertaken at each GP practice and the list generated by the search screened by the GP before invitation letters are sent out on behalf of the practice. Replies will be sent back to the research team who will then contact patients to organise their baseline appointment. To maximise recruitment a reminder letter, plus the study information pack, will be sent to patients who have not responded at all to the initial invitation. We will also advertise the study via media platforms accessed by people of South Asian ethnicity (radio, press, social media). The advert will advise that recruitment for the study is ongoing, provide information on the eligibility criteria, and invite individuals interested in participating to contact the study team (phone number and e-mail provided) to discuss participation in more detail.
Sample size	25
Registration/Randomisation:	Randomised controlled feasibility study.. Participants will be randomised to either commence the intervention immediately, or continue current treatment for T2DM for the next 3 months,

	prior to receiving the intervention.
Main Inclusion Criteria:	• Men and women aged 18-65 years<br>• South Asian ethnicities<br>• T2DM of duration 0-4 years<br>• Body mass index (BMI)>25 kg/m^2 and <45 kg/m^2
Main Exclusion Criteria:	• Current insulin use<br>• Recent routine HbA1c on record ≥12%<br>• Recent eGFR <30 mls/min/1.73^2<br>• Substance abuse<br>• Known cancer<br>• Myocardial infarction within previous 6 months<br>• Learning difficulties<br>• Unable to speak English<br>• Current treatment with anti-obesity drugs<br>• Diagnosed eating disorder or purging<br>• Pregnant/ considering pregnancy<br>• Patients who have required hospitalisation for depression or are on antipsychotic drugs<br>• People currently participating in another clinical<br>• research trial<br>• People with metal implants or devices and those who have claustrophobia cannot take part MRI scans are needed as part of the study
Product, Dose, Modes of Administration:	The intervention to be assessed is an 825-853kcal/day TDR, planned to be followed for 12 weeks, with stepped food reintroduction, using a tested energy-restricted nutritionally balanced (30% fat, <10% saturated fat) weight loss maintenance diet using food-group exchanges, plus support to increase physical activities to approach 15,000 steps/day if individually acceptable) with 30 minutes brisk-walking daily.<br>Both intervention and control arms will continue to receive standard care under current clinical guidelines for T2DM. The intervention will be delivered by research dietitians who are trained and competent in Counterweight Plus. Process evaluation will be conducted to assess the number of patients invited, the number who engage with the programme, and completion and attrition rates. Participants will be asked to give written feedback on the programme. Assessments of quality of life, weight loss expectations, and psychological process measures will be made. Mechanistic studies will also be conducted to investigate the changes in pancreatic and liver fat. Participants will be offered the option of undergoing measurements of MR and body composition<br>These data will inform a larger randomised controlled trial.
Duration of Treatment:	6 months
Statistical Analysis:	As this study is a feasibility study, this study is not powered to achieve statistical significance. Rather we are aiming to understand if we can<br>a) recruit men and women of South Asian origin to an intensive weight management intervention<br>b) if the intervention is acceptable to the South Asian population<br>c) if individuals of South Asian origin can lose weight and achieve diabetes remission with the intervention<br>d) how the programme can be improved for use in a larger randomised controlled trial<br>e) define the mechanisms behind Type 2 diabetes remission in the South Asian population

STUDY FLOW CHART

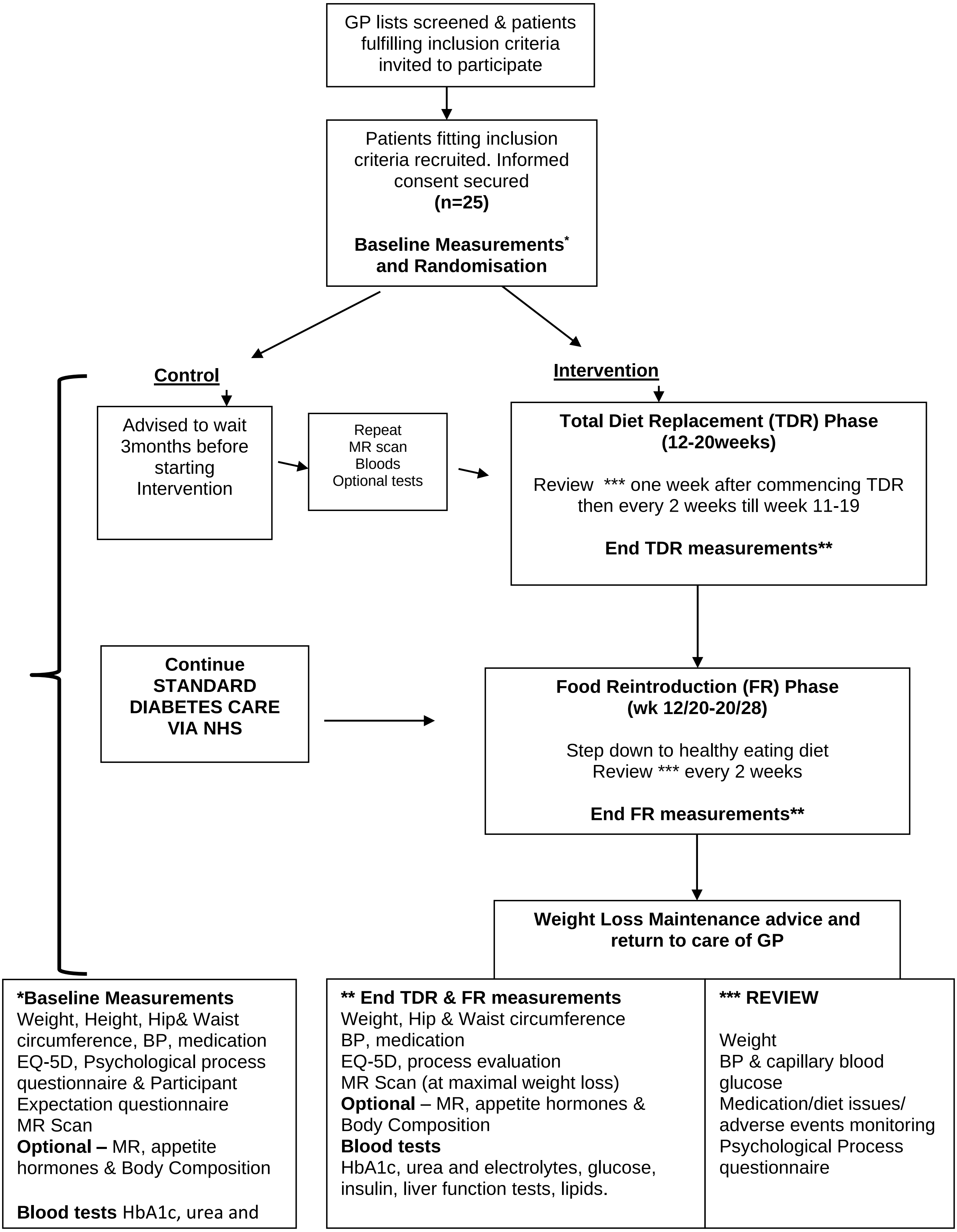

1. INTRODUCTION

1.1 Background: the problem

Age, genetics and being overweight or obese are the three key contributory factors to the development of T2DM.[1] Unlike age and genetic factors, people can affect their weight. Consensus now exists around weight loss in the region of 15kg (2-3stones) resulting in diabetes remission in a significant proportion of individuals with T2DM.[2] Around 1% of the Scottish population is of South Asian ethnicity. [3] South Asians are at higher risk of T2DM, developing the condition at a lower body weight than non-South Asians. [4] This coupled with evidence that South Asians are less likely to access weight management services than non-South Asians points to a need for novel approaches to manage this growing problem.[5] In the absence of appropriate management and support, the Scottish South Asian population would be at risk of developing health inequalities as well as the resulting health and health economic burden of sub optimal management of T2DM.[3] A programme initially focusing on a low energy liquid formula diet is showing promising results both in terms of acceptability, weight change and impact on HbA1c. [2,6,7] The formula food provides all essential nutrients in around 850kcal hence guarantees energy deficit and therefore weight loss with compliance. Many people appreciate the opportunity for a complete 'step away from food' and compliance is highly encouraging as compared with other weight management approaches due to the weight loss magnitude and associated clinical benefits such as reduction in fasting blood glucose and withdrawal of medications from as early as week one. The proposed study will examine if the initial phase of this approach using formula food products is acceptable to the South Asian population and if it results in similar clinical outcomes in terms of weight loss and diabetic status at the 3-6 month phase.

1.2 Rationale

Currently one in every hundred people in Scotland are of South Asian ethnicity and weight management and T2DM are two significant health issues. [3] However evidence suggests sub-optimal engagement and outcomes around weight management for the South Asian population.[8] The cost of diabetic medications in Scotland is currently around £80 million per year and has doubled in the last 10 years, largely related to obesity. [9] Figures therefore would suggest that just short of £1 million per year is spent on diabetic medications for the Scottish South Asian population of around 4500: NB this figure being conservative due to the higher risk of T2DM in the South Asian population. Without appropriate research and development in the South Asian population for this area of care, the cost of the burden of obesity will continue to multiply as well risking an increase in levels of health inequalities for this population.[3] There is overwhelming evidence that modest sustained weight loss, e.g. current target of 5-10% prevents the onset of most new T2DM in people with prediabetes and it improves all aspects of diabetes control (glycaemia, blood pressure, lipids and microvascular damage with reductions in drug doses.[10,11] Advice to lose and maintain 5-10% weight loss, by diet and exercise is included in most guidelines.[12] However, most T2DM patients are now managed in primary care, many do not see a dietitian, and few achieve 5% weight loss.[2]

1.3 Prior experience of managing obesity and type 2 diabetes

Our recent work has shown that a greater level of weight loss of around 15kg achieves reversal of diabetes and normal blood glucose control from day to day in a significant proportion of people with diagnosed T2DM.[2] Achieving this level of weight loss requires novel approaches such as use of formula products as part of a programme however the approach must be acceptable to the population being studied. The proposed study will assess if an existing evidence-based weight management programme, 'Counterweight-Plus', is acceptable to patients of South Asian origin and achieves weight change and T2DM as per non South Asian populations as compared with routine care. The primary focus is acceptability of TDR in this population and response in terms of weight change and remission of diabetes following the TDR. Initial findings will be used to inform a larger randomised controlled trial.

1.4 Study hypothesis

Losing weight using a structured weight management programme which includes an initial period of total diet replacement, followed by carefully managed food reintroduction and then weight loss maintenance, is a viable treatment for putting T2DM into remission, and can potentially be transferred to a larger scale as part of routine GP care, where large numbers of overweight people with T2DM are managed in the UK.

2. STUDY OBJECTIVES

The research will establish whether it is feasible and acceptable to offer patients of South Asian ethnicity an intensive weight management programme (known as Counterweight Plus) to achieve remission of diabetes. This study will provide data to support implementation of a larger randomised controlled trial aim at long term weight change in this population.

Research Questions

- Can patients of South Asian ethnicity be recruited into an intensive weight management programme (known as Counterweight Plus)?
- Do patients of South Asian ethnicity engage, adhere to and continue in the initial 2 phases of Counterweight Plus, achieve weight loss, diabetes remission and improve quality of life?
- What do patients of South Asian ethnicity like/dislike or advise can be improved with the initial 3 phases (screening, total diet replacement and food reintroduction) of Counterweight Plus intervention?

3. STUDY DESIGN

The study will follow a randomised controlled design with patients being randomised to either the weight management intervention or the control group. The control group will be asked to continue following usual management of type 2 diabetes for 3 months, prior to starting the intervention. This study will be performed according to

the Research Governance Framework for Health and Community Care (Second edition, 2006).

3.1 Study Population

25 patients will be recruited and randomised to either control or intervention. Patients will be recruited from general practices in Glasgow that have a high population of patients of South Asian origin. GP patient lists will be screened to identify patients, aged 18-65 years diagnosed with type 2 diabetes within the previous 2 years (ideally newly diagnosed), who would be potentially eligible to participate. GP's will review these lists and remove any patients they feel would be ineligible or unsuitable to approach.

3.2 Inclusion criteria

- Written informed consent
- Men and women aged 18-65 years
- South Asian ethnicities
- Body mass index (BMI)>25 kg/m^2 and <45 kg/m^2
- T2DM of duration 0-4 years (diagnosis based on a recorded diagnostic-level test: HbA1c and/or blood glucose)
- HbA1c $\geq$ 48 mmol/mol and/or fasting plasma glucose $\geq$ 7 mmol/l within last 12 months

3.3 Exclusion criteria

- Current insulin use
- Recent routine HbA1c on record ≥12%
- Weight loss of >5kg within the last 6 months
- Recent eGFR on record <30 mls/min/1.73^2
- Substance abuse
- Known cancer
- Myocardial infarction within previous 6 months
- Severe or unstable Heart Failure defined as equivalent to the New York Heart Association (NYHA):
  - Grade 3 - marked limitation of physical activity. Comfortable at rest, but less than ordinary activity causes fatigue, palpitation, or breathlessness, and
  - Grade 4 - unable to carry out any physical activity without discomfort. Symptoms of cardiac insufficiency at rest. If any physical activity is undertaken, discomfort is increased.

    or
  - Grade 2 if symptoms are not attributed to obesity - Slight limitation of physical activity. Comfortable at rest, but ordinary physical activity results in fatigue, palpitation, or breathlessness
- Learning difficulties
- Not fluent in English language
- Current treatment with anti-obesity drugs
- Diagnosed eating disorder or purging
- Pregnant/ considering pregnancy

- Patients who have required hospitalisation for depression or are on antipsychotic drugs
- People currently participating in another clinical research trial
- People with metal implants or devices and those who have claustrophobia cannot take part as MR scans are needed as part of the study

3.4 Identification of participants and consent

Prior to recruitment of patients, GP practices will be approached by letter to invite their participation. In participating GP practices potential participants will be identified by a computerised search of GP records. An experienced research nurse, with a current research passport will undertake this. As Caldecott Guardians, GP's will be asked for permission/approval for the research nurse to search their database. All data will remain within the practice.

Searches will be undertaken at each GP practice and the list generated by the search screened by GP's before the letters are sent out on behalf of the practice.

Patients identified by GP's as potentially eligible to participate in the study will be sent an invitation to participate on behalf of their GP. The invitation will include a written information sheet providing details of study. Should they wish, patients will be able to contact their GP for further discussion. Patients will be asked to respond to the researchers to indicate whether or not they are interested in participating. Reply paid envelopes will be provided.

Patients indicating an interest in participating will be invited to attend for a screening appointment where the study will again be fully explained and discussed. Patients will be reassured that participation is voluntary and that withdrawal at any point would not affect their routine care. Patients fulfilling the inclusion criteria will then be asked to give their written consent to participate in the study and for clinical and outcome data to be extracted from GP records for up to 1 year from their initial appointment. These data will be extracted by the study research nurse. The consent form will be signed by both the patient and the research dietitian.

To maximise recruitment a reminder letter, plus the study information pack, will be sent to patients who have not responded <u>at all</u> to the initial invitation after 2 weeks.

We will also advertise the study via media platforms accessed by people of South Asian ethnicity (radio, press, social media). The advert will advise that recruitment for the study is ongoing, provide information on the eligibility criteria, and invite individuals interested in participating to contact the study team (phone number and e-mail provided) to discuss participation in more detail.

3.4.1 Non respondents

A screening log will be set up to record age, sex, BMI and duration of diabetes of all patients from the GP lists who are identified by the research nurse as satisfying the recruitment criteria for STANDby, and invited to participate. An anonymised list from

each GP practice detailing the age, sex, height and weight (to allow calculation of BMI), duration, or date of diagnosis, of diabetes and deprivation score (derived from postcode) of patients invited to participate, will be recorded. These data will be compared with the same data from patients who agree to participate in the study. This will allow reporting of the total number of patients invited to participate, the characteristics of those who decline, or do not respond to the invitation to participate, and determine differences, if any, between those who participate and those who do not. Such analyses are important to identify any recruitment bias and determine the representativeness of the study participants.

3.5 Withdrawal of subjects

Patients who withdraw from the trial protocol will continue to have data collected from their routine diabetic clinic/GP visits, for up to 12 months from their initial appointment, unless they specifically withdraw consent for this. Data analysis will use best available follow-up weights (from routine attendances) and end of study diabetes status for subjects who discontinue the formal weight management programme. Drug intolerance, diet intolerance or poor-compliance will be recorded.

4 Trial procedures

Patients will all be seen at the Glasgow Clinical Research Facility for all appointments.

Patients randomised to control will be advised to continue with usual care for 3 months before coming back to receive the intervention. They will be asked not to aim to lose any weight during the initial control period, but rather to wait until they commence the intervention part of the study. An appointment will be given to the patient for 3-months' time, and they will commence the intervention at that point.

Patients randomised to intervention will commence Counterweight Plus, which includes a TDR phase followed by structured food reintroduction. This will be delivered by research Dietitians experienced in programme delivery.

TDR phase (0 to 12-20wks): A commercial micronutrient-replete 825-853kcal/d LELD will be provided (Cambridge Weight Plan) to replace normal foods, with ample fluids, for 12-20 weeks. Oral hypoglycaemic agents (OHA), antihypertensive and diuretic drugs will be withdrawn on commencement of TDR, and reintroduced (according to guidelines) if diabetes or hypertension returns. Aspirin will be continued in patients prescribed this because of a previous MI (prior to the previous 6 months), but will be discontinued in patients who are prescribed it purely because they have T2D. Beta-blockers will be continued in patients prescribed these for the management of angina. A soluble fibre supplement (Fybogel 2x3.5g/day) will be prescribed to avoid constipation.

Participants will return for review one week after commencement on the TDR and at 2 weekly intervals thereafter till week 12-20 (total of 7-13 appointments) and the commencement of the food reintroduction stage.

To allow some flexibility for patients whose commitments, or practical limitations vary, the TDR phase will be permitted to continue up to 20 weeks if they fail to lose >15kg by week 12. Any patient whose BMI falls below 22 kg/m^2, before week 12 will be moved forward to 'food reintroduction and weight maintenance**.** Reasons for moving to food reintroduction prior to week 12 will be recorded.

Food Reintroduction (FR) phase (weeks 12-18):
A stepped transition to food based Weight Maintenance, replacing TDR with meals which contain 30% of energy from fat. Some further modest weight loss will occur.
Week 13: step down to 400kcal/d LELD + 1 low-fat meal/day (c. 360-400 kcal) + 2 servings of fruit, 200mls skimmed milk and free vegetables. Total intake will be 1000kcal/day.
Week 15: step down to 200kcal/d LELD + 2 low-fat meals/day (c. 720-800 kcal) + 2 servings of fruit, 200mls skimmed milk and free vegetables. Total intake will be 1200kcal/day.
Week 17: 3 low-fat meals per day (c.1080-1200 kcal) + 2 servings of fruit, 200mls skimmed milk and free vegetables. Total intake will be 1400kcal/day.
Week 18: 3 low-fat meals per day (c.1080-1200 kcal) + 2 servings of fruit, 200mls skimmed milk and free vegetables. Advice to increase by 200kcal per day until weight loss maintenance achieved. Option to join BEYOND Study.

During this phase participants will attend for review appointments every 2 weeks. To allow flexibility for patients whose confidence varies, the food introduction phase will be permitted to be varied between the protocol-defined limits of 2-8 weeks before switching to full food-based weight maintenance. Reasons for moving to weight loss maintenance without completing food reintroduction will be recorded. If patients are unable to attend appointments, they will be offered telephone consultation as necessary but with encouragement to attend clinics as the preferred method of contact.

During the period of restrictions due to COVID-19

- Participants will not be seen in person for study appointments. Appointments/consultations will be carried out, by, the study research dietitian, by telephone/text/e-mail, whichever is preferred by the participant.

On completion of the feasibility study, at the end of FR we will provide participants with advice, and written information, on maintaining weight loss, which will include goal setting, self-monitoring, physical activity, relapse prevention if weight tends to rise again, and education on food labelling and eating out. Participants will then be returned to the care of their GP, who will be informed

Outcome Measurements – All participants
Baseline measurements (App A), with the exception of height, will be repeated at the end of FR. All measurements will be recorded by the research dietitians.

Height will be measured to the nearest mm, with the Frankfort plane horizontal, using a portable stadiometer (Chasmors Ltd, London).

Body weight will be measured to the nearest 100g in light clothing without shoes using Class 111 approved calibrated scales. The scales will provide a printout of the participant's weight, which will be photocopied and stored securely

MR scans will be carried out at baseline (prior to weight loss) and repeated at the point of maximal weight loss. Participants allocated to an initial 3-month control period will have an additional MR scan carried out at the end of the control period, prior to starting the weight management intervention. Scans will be carried out at the Clinical Research Imaging Facility, Queen Elizabeth University Hospital, Glasgow. Anonymised digital data derived from the scanning procedure, will be sent by secure transfer to Advanced MR Analytics (AMRA) for analysis of fat content and distribution.

Optional Tests:
Measurement of Metabolic Rate (resting and during physical activity), appetite hormones and Body Composition (App B)

Participants will be asked to consider undergoing additional tests to measure Metabolic Rate, appetite hormones and Body Composition. These optional measurements will be taken at baseline, end of TDR and end of study and will be carried out in the Metabolic Laboratory, New Lister Building, Glasgow Royal Infirmary

Due to the restriction imposed by the COVID-19 pandemic it has not been possible to continue with these tests. On recommencement of recruitment these tests will not offered to potential participants.

4.1 Study schedule (App A)

Appointment (Appt) 0: Baseline (All Patients)

- Review and discuss study participation
- Review inclusion/exclusion criteria including readiness to change screening
- Secure informed consent
- Assign study ID
- Measurement of: height, weight, waist & hip circumference & blood pressure
    - Weight measured using study scales and printout of weight countersigned by practice nurse/dietitian and CW specialist
    - Three blood pressure readings recorded 2-3 minutes apart
    - Waist and hip not measured if BMI>35kg/m^2

Venepucture for:

- HbA1c, urea and electrolytes, glucose, insulin, liver function tests, lipids.)*. All Fasting Sample, as per Sample Handling Manual (App C)
- Pregnancy test in women of childbearing years
- Questionnaire completion: EQ-5D, Participant Expectation and Psychological Process Questionnaire
- Randomise patient to CONTROL or INTERVENTION
- Completion of practitioner workbook by research dietitians
- Appointment made for MRI Scan

- Arrange appointment for MR, body composition and appetite hormones measurements if participant has consented to these.

- **INTERVENTION ONLY**: Samples of TDR products given to patients to allow choice of product to be used during intervention to be made.
    - **CONTROL ONLY**: Advised to continue with usual care as per current guidelines. Appointment made for 3 months for repeat
    - measurement of MR, body composition and appetite hormones,
    - MRI scan and venepuncture for HbA1c, urea and electrolytes, glucose, insulin, liver function tests,
    - commencement of weight management intervention

Appt 1: Week 0+1
Intervention Participants

- Measurement of weight, blood pressure and capillary blood glucose (measured by blood glucose meter) recorded
- Oral hypoglycaemic, antihypertensive and diuretic medications discontinued
    - Aspirin only discontinued in patients who are prescribed it purely because they have T2D.
    - Beta-blockers continued in patients prescribed these for the management of angina.
- Commence TDR phase of intervention as per Counterweight Plus Appointment Planner (App C)
    - Fybogel supplement prescribed
- Appointment made for next visit

Appt 2-7(Weeks 0+2 – 12)

- Measurement of weight & blood pressure
- Capillary blood glucose measured by blood glucose meter
- Psychological Process Questionnaire
- Delivery of TDR intervention as per Counterweight Plus Appointment Planner (App D)
- Waist circumference measured (if appropriate) at visit 7 (wk0+12)
- Venepuncture for HbA1c, urea and electrolytes, glucose, insulin, liver function tests, lipids.at week 12/end of TDR phase
- MR, appetite hormone and Body Composition measurements at week 12/end of TDR phase if participant has consented to these

Appt 8, 9, 10 (Weeks 0+13-17)

- **Appt 8 only** ask GP to repeat HbA1c -
- Measurement of weight & blood pressure
- Capillary blood glucose measured by blood glucose meter
- Psychological Process Questionnaire
- Food reintroduction phase intervention as per Counterweight Plus Appointment Planner (App C)

Appt 11 (Week 0+19, Final Appointment)

- Venepuncture for HbA1c, urea and electrolytes, glucose, insulin, liver function tests, lipids.
- Measurement of weight & blood pressure
- Measurement of waist and hip circumference
- Psychological Process Questionnaire
- EQ-5D
- Process evaluation questionnaire
- MR, appetite hormone and Body Composition measurements if participant has consented to these

Final MR scanning will be undertaken at the point of maximal weight loss which will be either at the end of the TDR phase or end of food reintroduction

On completion of the study participants with be given advice, and written information, on maintaining weight loss, which will include goal setting, self-monitoring, physical activity, relapse prevention if weight tends to rise again, and education on food labelling and eating out. Participants will then be returned to the care of their GP, who will be informed.

4.2 Study Outcome Measures

The main study outcome measures are as follows:

- Numbers recruited into the intervention
- Programme retention
- The acceptability of the intervention to people of South Asian origin
- Weight loss achieved (kg, %), and diabetes remission. Remission is defined as a return of HbA1c to <48mmol/mol (<6.5%) that occurs spontaneously or following an intervention, and off all usual glucose-lowering pharmacotherapy for at least 3 months
- Define the mechanisms behind type 2 diabetes remission in the South Asian population

4.3 Laboratory Tests

All participants will have blood samples **(App D)** taken at baseline, end of the Total Diet Replacement phase, and on completion of study for:
Routine Biochemistry - Liver Function tests, urea and electrolytes, cholesterol, triglycerides, high density lipoproteins: HbA1c and glucose. Additional analyses will be carried out to explore changes in insulin, hsCRP, Ferritin, Urate and creatinine. Additional tests may be added, if indicated by new information, using the existing samples, to add information about safety or pathophysiology of T2D in people of South Asian origin.

ll blood samples will be processed by, Central Glasgow laboratory (BHF GCRC, University of Glasgow).

GP's will be asked to repeat participants HbA1c at the end of the Total Diet Replacement phase and end of the study, as part of routine NHS care, to reassess diabetic status and establish their on-going treatment needs. These samples will be processed in an NHS laboratory.

During the period of restrictions due to COVID-19

- Participants will be asked to attend Glasgow Clinical Research Facility on completion of TDR and at the end of FR to have blood samples for HbA1c taken and blood pressure checked. These measurements will be taken by CRF nursing staff. Should the NHS CRF facilities and staff at GRI not be available, the necessary follow-up checks will be carried out by Professor Mike Lean (PI) at the Human Nutrition Metabolic Room in the New Lister Building.
- Blood samples will be processed by NHS GG&C laboratories and results accessed via Portal by the research dietitian. GPs will be informed of the results and the need for recommencement of medications discussed as required.

5. ASSESSMENT OF SAFETY

Given the nature of the intervention there is very low likelihood of safety concerns. Patients allocated to the intervention will be closely monitored throughout the study with review appointments 2 weekly during the TDR and Food Reintroduction phases. At each appointment blood pressure and postural symptoms will be monitored and antihypertensive therapy reintroduced if necessary according to clinical guidelines. ***Appendix E***

Patients will be given clear guidance on detecting deteriorating glycaemia to permit hypoglycaemic agents to be reintroduced if necessary. If capillary blood glucose is ≥20mmol/l at any appointment we will ask the participants GP check the participants HbA1c. Results will be discussed with the participants GP to allow decisions on changes to medications to be made.

Any observations/results which may pose a risk to health will be discussed with the patient and their GP.

6. STATISTICS AND DATA ANALYSIS

The main objective of the study is to investigate if Counterweight Plus is acceptable to individuals of South Asian origins. This study is not powered to achieve a primary outcome, rather to investigate if it is possible to recruit, retain and achieve remission from diabetes in the South Asian population. Data will be collected on paper-based forms and entered into a secure SPSS file for analysis. Drug intolerance, diet intolerance or poor-compliance will be recorded. Trial documentation and data will be retained for at least 5 years.

6.1 Sample size

We aim to recruit 25 participants to the study. This figure has been chosen as this will give us enough information to assess the feasibility and acceptability of the programme to this population. We anticipate a failure to complete rate of about 25% (this may be greater at specific timepoints due to the COVID-19 pandemic). With 20 participants in a randomized comparison, the study can estimate the change in weight with a margin of error of 2.8kg assuming a standard deviation weight change over 3 months of 6kg. e.g., for an average observed weight change of 8kg (SD 6) we can estimate with 95% confidence that in the population the true weight change would lie between 5.1 and 10.7kg.

The proportion achieving remission in the combined intervention and delayed intervention groups (n=20) will be compared to the proportion achieving diabetes remission in the external DIADEM-1 trial control group (Taheri et al, Lancet Diabetes and Endocrinol, 2020) (12%; 95%CI 5%, 21%). Therefore assuming 12% remission in a usual care group (based on DIADEM), using a one sample proportion test, with 20 participants there will be 83% power to detect a significant difference if 30% of participants (n=6) or more achieve remission.

6.2 Management and delivery

The co-investigators will manage and analyse trial data. All statistical analyses will be conducted according to a pre-specified Statistical Analysis Plan.

7.0 STUDY CLOSURE / DEFINITION OF END OF STUDY

The trial will last for 13 months in total. All participants will have completed 6 months follow-up by the end of the first year. This will give 6months to recruit all participants to the study.

8. Data Handling

The study will follow a randomised controlled design. A blinded envelope system will be used for treatment allocation. The randomisation sequence will be produced by an independent researcher from the University of Glasgow using an online number generation tool. Envelopes will be prepared by the same researcher who will play no part in recruitment, treatment allocation or delivery of the intervention.

8.1 Case Report Forms / Electronic Data Record

Data will be entered into the practitioner workbook, then transferred into an SPSS data file. This will be stored securely on the University of Glasgow drive. Access will only be possible for the named applicants of this study and study sponsors for audit purposes.

8.2 Record Retention

Data will be held for a minimum of 5 years

9. STUDY MONITORING/AUDITING

This study research team will monitor recruitment to ensure the required numbers are recruited to the study in the time frame.

Adverse Event Monitoring
Enquiry at each study visit will identify the occurrence of adverse, or events of interest and these will be recorded **(App F)**.In the case of any serious adverse event occurring, the PIs of the study will be informed immediately, and these managed with guidance from the PIs. The study sponsor and REC will also be informed of any serious adverse events.

10. PROTOCOL AMENDMENTS

Any change in the study protocol will require an amendment. This will be discussed with Sponsor and submitted to REC.

11. ETHICAL CONSIDERATIONS

The study will be carried out in accordance with the World Medical Association Declaration of Helsinki (1964) and its revisions (Tokyo [1975], Venice [1983], Hong Kong [1989], South Africa [1996] and Edinburgh [2000]).
Favourable ethical opinion will be sought from an appropriate REC before patients are entered into this clinical trial. Patients will only be allowed to enter the study once either they have provided written informed consent. The PI will be responsible for updating the Ethics committee of any new information related to the study.

12. INSURANCE AND INDEMNITY

The South Asian Diabetes Remission Feasibility Study is sponsored by NHS Greater Glasgow & Clyde. In this case the Sponsor will be responsible for negligent harm due to the management/conduct of the study through the Clinical Negligence and Other Risks Indemnity Scheme (CNORIS). Harm from the design of the study will be covered by the University of Glasgow's clinical trials insurance. The NHS has a duty of care to patients treated, whether or not the patient is taking part in a clinical trial, and the NHS remains liable for clinical negligence and other negligent harm to patients under its duty of care.

13. FUNDING

FUNDER:

Department of Human Nutrition, University of Glasgow
Department of Metabolic Medicine, University of Glasgow

14. ANNUAL REPORTS

Annual progress reports will be submitted as directed by the study sponsor.

15. REFERENCES

1. Taylor R. Type 2 Diabetes Etiology and reversibility. *Diabetes Care* 2013; **36**(4): 1047-55.
2. Leslie WS, Ford I, Sattar N, et al. The Diabetes Remission Clinical Trial (DiRECT): protocol for a cluster randomised trial. *Bmc Family Practice* 2016; **17**.
3. Sattar N, Gill JMR. Type 2 diabetes in migrant south Asians: mechanisms, mitigation, and management. *Lancet Diabetes & Endocrinology* 2015; **3**(12): 1004-16.
4. Excellence. NIoC. Assessing body mass index and waist circumference thresholds for intervening to prevent ill health and premature death among adults from black, asian and other minority ethnic groups in the UK. Manchester: NICE; 2013;.
5. Wallia S, Bhopal RS, Douglas A, et al. Culturally adapting the prevention of diabetes and obesity in South Asians (PODOSA) trial. *Health Promotion International* 2014; **29**(4): 768-79.
6. Lean M, Brosnahan N, McLoone P, et al. Feasibility and indicative results from a 12 month low-energy liquid diet treatment and maintenance programme for severe obesity. *British Journal of General Practice* 2013; **63**(607): E115-E24.
7. Leslie W, Taylor R, Lean M. Weight Loss with Liquid Formula Diets in Obese Patients with and without Diabetes. *Faseb Journal* 2015; **29**.
8. Mukhopadhyay B, Forouhi NG, Fisher BM, Kesson CM, Sattar N. A comparison of glycaemic and metabolic control over time among South Asian and European patients with Type 2 diabetes: results from follow-up in a routine diabetes clinic. *Diabetic Medicine* 2006; **23**(1): 94-8.
9. Division NSIS. Prescribing & Medicines: Reimbursement and remuneration paid to dispensing contractors. Quarter Three of Financial Year 2015/16.
10. Pownall HJ, Bray GA, Wagenknecht LE, et al. Changes in Body Composition Over 8 Years in a Randomized Trial of a Lifestyle Intervention: The Look AHEAD Study. *Obesity* 2015; **23**(3): 565-72.
11. Leeds AR. Formula food-reducing diets: a new evidence-based addition to the weight management tool box. *Nutrition Bulletin* 2014; **39**(3): 238-46.
12. NICE. NICE. Obesity: The Prevention, Identification, Assessment and Management of Overweight and Obesity in Adults and Children.London: NICE; 2006.

APPENDIX A: STANDby SCHEDULE OF ASSESSMENTS

		Total Diet Replacement Phase							Food Reintroduction Phase			
**Appointment**	**0**	**1**	**2**	**3**	**4**	**5**	**6**	**7**	**8**	**9**	**10**	**11**
**Week**	**0 (Baseline)**	**0+1**	**0+2**	**0+4**	**0+6**	**0+8**	**0+10**	**0+12**	**0+13**	**0+15**	**0+17**	**0+19**
**Study Procedure**												
Review & discuss study participation	√[a]											
Review Inclusion/Exclusion Criteria	√[a]											
Obtain Informed Consent	√[a]											
Height	√[a]											
Weight	√	√	√	√	√	√	√	√	√	√	√	√
Waist Circumference	√							√				√
Hip Circumference	√											√
Blood Pressure	√	√	√	√	√	√	√	√	√	√	√	√
Blood samples[c]	√							√				√
Capillary blood glucose		√	√	√	√	√	√	√	√	√	√	√
Repeat HbA1c									√			√
EQ-5D questionnaire	√											√
Participant Expectation Questionnaire	√[a]											
Psychological Process Questionnaire	√		√	√	√	√	√	√	√	√	√	√
MR Scanning[c]	√											√
MR Measurements [b]	√								√			√
Body Composition Measurements [b]	√								√			√
Appetite hormones [b]	√								√			√

[a] initial visit only
[b] optional measurements
[c] Control participants only – Bloods, MRI scan & optional measurements repeated at end of control period

Appendix B: Measurement of Metabolic Rate, appetite hormones and Body Composition

Resting and non-resting metabolic rate and appetite hormone measurements

Metabolic rate will be measured by indirect calorimetry in the fasted state and after breakfast by means of computerised open-circuit ventilated hood system (Quark RMR, Cosmed, Italy). For non-resting metabolic rate, the participant will undertake a low intensity walking test on a treadmill (Trackmaster Treadmills, Full Vision, Inc., Kansas, USA) during which intensity will be gradually increased from 2 km/h to 5 km/h. The whole test is expected to last no longer than 20 minutes. Blood samples will be taken before and after breakfast to measure gut appetite hormones (PYY, GLP1, CCK), leptin and insulin.

Anthropometric and Body Composition Measurements

Height will be measured to the nearest 0.5 cm using a stadiometer (Seca, Leicester, UK). Body mass and body fatness will be measured using leg-to-leg bioelectrical impedance scales (TBF-300, TANITA, Cranela, UK) (Androutsos et al., 2015). Waist circumference will be measured using a non-elastic tape measure.

Adaptive reduction in thermogenesis

Adaptive reduction in thermogenesis will be calculated as the differences between measured and predicted metabolic rate. As described above, measured values will be obtained by indirect calorimetry. Predicted values will be calculated using regression equations incorporating fat mass and fat free mass (as well as age and sex), and anthropometric measurements (height, weight).

Participants will attend the metabolic laboratory at the New Lister Building, Glasgow Royal Infirmary for the above measurements which will be carried out by trained and experienced research staff.

.

Appendix C: Counterweight Plus Programme Appointment Planner

APPOINTMENT PLANNER

TOTAL DIET REPLACEMENT (APPOINTMENT 1)	
**Resources**	**Practitioner tasks and discussion points**
Provide the COUNTERWEIGHT PLUS Total Diet Replacement Folder:<br><br>Welcome to COUNTERWEIGHT PLUS<br><br>**Weight Tracker**<br><br>**Getting Ready**<br><br>**Daily Living Diary**<br><br>**Managing Difficult Times**<br><br>**Total Diet Replacement Plan**	Welcome to COUNTERWEIGHT PLUS<br>Discuss programme stages and support. Individual and Practitioner to sign the Promise<br>Weight Tracker<br>Measure and record weight and waist circumference on weight tracker and data collection system<br>Getting Ready<br>Discuss the following:<br>- advantages and disadvantages<br>- dealing with disadvantages<br>- weight history, previous weight loss attempts, weight loss expectations<br>- set initial 5% weight loss target, and advise on weekly weight loss target<br>- self monitoring of weight at home Total Diet Replacement Plan Discuss and plan for:<br>- a typical day's intake on 800+ Calories Plan including fluid intake<br>- preparing shakes and soups and important considerations<br>- possible side effects, causes and what actions to take, and other considerations. Prescribe fibre supplement as appropriate<br>Ensure a minimum of I weeks supply of shakes and soups is available - consider flavour preferences and use sachet table to calculate quantity of provision<br>Daily Living Diary<br>Discuss importance of diary keeping. Advise to keep diary for first 2-3 days of the Total Diet Replacement Plan. Highlight the value of<br>recording side effects and bowel habits<br>Managing Difficult Times<br>Discuss the following:<br>- Stop,Think, Learn and Plan technique<br>- High risk situations and alternative activities<br>- Planned and unplanned time off the Total Diet Replacement Plan<br>- Written plan for planned time off<br>- Social support<br>Weight Tracker<br>Agree next appointment and record on tracker. Record Counterweight Practitioner name, contact telephone number and advise on best contact method

TOTAL DIET REPLACEMENT (APPOINTMENT 2)	
Resources	Practitioner tasks and discussion points
	Weight Tracker<br>Measure and record weight on Weight Tracker and data collection system<br>Getting Ready<br>Review weight loss expectations, and targets. Amend target if necessary<br>Total Diet Replacement Plan<br>Discuss progress and compliance with Total Diet Replacement Plan<br>Review side effects, activity, tolerance to fibre supplement and bowel habits<br>Ensure 3 weeks shakes and soups available - consider flavour preferences and use sachet table to calculate provision<br>Daily Living Diary and Managing Difficult Times<br>Review use of sections for self monitoring and relapse prevention strategies<br>Weight Tracker<br>Agree next appointment and record on tracker. Highlight importance of weekly self weighing
**TOTAL DIET REPLACEMENT (APPOINTMENTS 3,4,5,6,7)**	
	Weight Tracker<br>Measure and record weight on Weight Tracker and data collection system<br>At Appointment 7<br>- Calculate BMI and measure waist circumference<br>- If >15kg weight loss is not achieved continue on Total Diet Replacement Plan up to 20 weeks<br>- If weight loss >15kg *NOTE: Only 1week between Appointment 7 and 8*<br>Review Getting Ready<br>Review weight loss expectations, and targets. Amend target if necessary<br>Total Diet Replacement Plan<br>Discuss progress and compliance with Total Diet Replacement Plan<br>Review side effects, activity, tolerance to fibre supplement and bowel habits<br>Ensure supply of shakes and soups - consider flavour preferences and use sachet table to calculate provision<br>Managing Difficult Times and Daily Living Diary<br>Review use of sections for self monitoring and relapse prevention strategies<br>Weight Tracker<br>Agree next appointment and record on tracker. Highlight importance of weekly self weighing

© Counterweight Ltd.

FOOD REINTRODUCTION PLAN (APPOINTMENT 8)	
**Resources**	**Practitioner tasks and discussion points**
Provide **COUNTERWEIGHT PLUS** Food Reintroduction Folder:<br><br>**Weight Tracker**<br><br>**Getting the Balance Right**<br><br>**Food Reintroduction Plan**<br><br>**Getting Active**<br><br>**Advice for People Taking Orlistat**<br>*NOTE: only applies to Rescue Plan patients*	Weight Tracker<br>Measure and record weight on weight tracker and data collection system<br>Getting Ready, Managing Difficult Times and Daily Living Diary<br>Review weight loss expectations and amend targets if necessary. Review relapse prevention and self monitoring strategies<br>Getting the Balance Right and Food Reintroduction Plan<br>Discuss eatwell plate. Outline the process of introducing one meal at a time. Discuss details of 1000 Calories Plan, how to introduce a meal (360-400calories) and the additional foods and drinks that can be taken Discuss serving size and calorie information.<br>Use the Food Reintroduction section to plan a day's intake<br>Getting Active<br>Discuss section. Review barriers and strategies to being more active. Make an activity SMART Goal<br>Weight and Waist Tracker<br>Agree next appointment and record on tracker
FOOD REINTRODUCTION PLAN (APPOINTMENTS 9,10)	
Discuss 2 of the following sections:<br><br>**Confused about Food Labels?**<br><br>**Shopping, Cooking and Eating Out**<br><br>**Alcohol and Weight**<br><br>**Eating Behaviours**	Weight Tracker<br>Measure and record weight on weight tracker and data collection system<br>Getting Ready, Managing Difficult Times, Daily Living Diary, Getting Active<br>Review weight loss expectations and amend targets if necessary. Review relapse prevention and self monitoring strategies<br>Food Reintroduction Plan and Getting the Balance Right<br>Review progress and compliance. Provide details of 1200 Calories Plan (Appointment 9) or 1400 Calories Plan (AppointmentI0) Discuss the amounts and types of food for meals. Use the Food Reintroduction section to plan a day's intake<br>Discuss two of the following sections:<br>Confused about Food Labels?, Shopping Cooking and Eating Out, Alcohol and Weight, Eating Behaviour<br>Review section and set a SMART Goal if appropriate<br>Weight and Waist Tracker<br>Agree next appointment and record on tracker

© Counterweight Ltd.

FOOD REINTRODUCTION (APPOINTMENT 11)	
**Resources**	**Practitioner tasks and discussion points**
**Your Personal Calories Plan**<br><br>**Snack Attack**<br><br>**Goal Setting**	Weight Tracker<br>Measure and record weight on weight tracker and data collection system<br>Getting Ready, Managing Difficult Times, Daily Living Diary, Getting Active<br>Review weight loss expectations and amend targets if necessary. Review relapse prevention and self-monitoring strategies<br>Confused about Food Labels?, Shopping, Cooking and Eating Out, Alcohol and Weight, Eating Behaviour<br>Review sections and SMART goals<br>Food Reintroduction and Getting the Balance Right<br>Review progress and compliance<br>Your Personal Calories Plan<br>Discuss achieving a stable weight and preventing weight regain. Calculate Personal Calories Plan for individual. Highlight adding<br>200 calories daily to the Plan each week for weight stabilisation<br>Snack Attack<br>Highlight snack allowance and hunger scale<br>Goal Setting<br>Discuss SMART approach and information in the Getting Active and Daily Living Diary section. Set 2-3 goals if appropriate<br>Weight and Waist Tracker<br>Agree next appointment and record on tracker

© Counterweight Ltd.

APPENDIX D: Sample Handling Manual

1. Blood Collection

Purpose

The purpose of this Sample Handling Manual is to describe the procedures to collect and process the blood samples for the STANDby study

Procedure

- Set out a disposable white tray with the following:-
  labelled blood sample bottles (see below)
  alcohol wipe
  gauze swab
  plaster
  vacutainer holder
  butterfly needle
  tape

- Explain procedure to patient

- Ensure patient is sitting/lying comfortably with their arm supported

- Attach needle to vacutainer holder

- Apply tourniquet to patient's upper arm and palpate to find suitable vein

- Wash hands or use alcohol hand scrub. Clean patient's arm with alcohol wipe

- Allow to dry before inserting needle

Insert needle into vein and attach vacutainer bottle to holder.

Biochemistry Samples

To avoid cross contamination between sampling tubes, the blood samples should be taken in the following order:

	Type of tube	Colour (indicative)	Volume of tube	Test
1	SST Serum separator	Yellow top	8.5ml	• U&Es<br>• LFTs<br>• Cholesterol<br>• triglyceride<br>• HDLc
2	EDTA	Purple top	4ml	• HbA1C
3	Fluoride Oxalate	Grey top	2ml	• Glucose

****The exception to this sequence would be if there was difficulty or concerns with poor venous access or if a different blood collection system is required for routine test samples which makes this sequence impractical. In this case, priority should be given to collection of the 4ml plasma sample for HbA1C measurement and the fluoride oxalate sample (primary study outcomes)***

- Slacken tourniquet once good blood flow is achieved
- Allow at least 10 seconds for a complete blood draw into each tube. Ensure blood has stopped flowing into the tube before removal
- Once full sample has been taken, remove the tourniquet
- Cover puncture site with gauze swab and remove needle from arm**.** **After** needle is removed apply pressure to area
- Instruct patient to keep their arm straight and press firmly for several minutes
- All sample bottles should be gently inverted 8 times to ensure thorough mixing
- Dispose of used needle, swab and vacutainer holder in sharps bin
- Dispose of swab in clinical waste bag
- Check patient's arm and ensure puncture site has stopped bleeding. Check if patient is taking anticoagulant medication
- Ask patient if they are allergic to plaster. If not, apply to puncture site

Any concerns/adverse events during or immediately after the procedure, please complete an adverse event form and follow up until resolved.

Processing of samples

- All blood samples will be transported to the Central Glasgow laboratory (BHF GCRC, University of Glasgow) by the Research Dietitian, where they will be processed and analysed.
  In the event that a sample cannot be brought on the same day the RA should contact the lab for advice. On receipt of samples, the samples will be processed by technicians on the same day of arrival.

Appendix E: Protocol for management of blood pressure

Background

Antihypertensive and diuretic drugs will be stopped on the day the total diet replacement is commenced. This is a safety measure, because blood pressure is likely to fall on the diet.

This protocol lays out the standard approach to be followed to allow clear description of the research findings. Individual clinical decisions may be necessary for a person's best interest.

The level of 140mmHg is chosen to allow safe decisions during the weight loss period. After the Food Reintroduction period follow usual guidelines for management of hypertension.

To simplify decision making, systolic pressure only is used as a guide to therapy even though both systolic and diastolic are relevant to long term benefit.

Protocol

When antihyperensive drugs are stopped, re-emphasise the importance of avoiding sodium (salt)

1. In the first 2 weeks after stopping antihypertensives and diuretics:
   If systolic BP over 165 mmHg on repeated measurement - restart one drug, as below.
2. Thereafter, if systolic BP is >140 mmHg - restart one drug as below.
3. Increase dose weekly to achieve target.
4. If systolic BP remains >140mmHg on the first drug - add a second drug, as below.
5. Increase dose weekly to achieve target.
6. Repeat as necessary with third, fourth or more drugs (increasing each to maximum dose).

Order of reintroduction of previously used drugs

1. ACE inhibitors (ramipril. lisinopril, perindropril , etc)
2. Angiotensin receptor blockers (irbesartan, candesartan etc)
3. Thiazide type (bendroflumethazide, indapamide etc)
4. Spironolactone
5. Calcium channel blocker (nifedipine, amlodipine etc)
6. Beta blocker (atenolol, labetolol etc)
7. Alpha blocker (doxazosin, prazosin)
8. All others

Appendix F: Events of Special Interest ,Medical events and Hospitalisation

If patient has any Events of Special Interest use the grading system below to grade the symptom severity.

Grading System
SINCE LAST STUDY APPOINTMENT
0 = not present
1 = mild (no real interference with daily activities)
2 = moderate (occasional/minor interference with daily activities)
3 = severe (frequent/constant/marked interference with daily activities)

		Total Diet Replacement Phase							Food Reintroduction Phase			
Appointment<br>Week	0<br>0<br>(Baseline)	1<br>0+1	2<br>0+2	3<br>0+4	4<br>0+6	5<br>0+8	6<br>0+10	7<br>0+11	8<br>0+12	9<br>0+14	10<br>0+16	11<br>0+18
Date												
Nausea												
Diarrhoea												
Constipation												
Dizziness												
Heartburn/Indigestion												
Headache												
Mood changes												
Sensitivity to cold												
Hair loss												
Fatigue												

If the patient has any medical contacts or is hospitalized, record details below

Date	Medical contacts since last study appointment (Details)	Hospitalisations since last study appointment (Details)

Date	Medical contacts since last study appointment (Details)	Hospitalisations since last study appointment (Details)

Date	Medical contacts since last study appointment (Details)	Hospitalisations since last study appointment (Details)
